# Validation of a Paralimbic-Related Subcortical Brain Dysmaturation MRI Score in Infants with Congenital Heart Disease

**DOI:** 10.3390/jcm13195772

**Published:** 2024-09-27

**Authors:** William T. Reynolds, Jodie K. Votava-Smith, George Gabriel, Vincent K. Lee, Vidya Rajagopalan, Yijen Wu, Xiaoqin Liu, Hisato Yagi, Ruby Slabicki, Brian Gibbs, Nhu N. Tran, Molly Weisert, Laura Cabral, Subramanian Subramanian, Julia Wallace, Sylvia del Castillo, Tracy Baust, Jacqueline G. Weinberg, Lauren Lorenzi Quigley, Jenna Gaesser, Sharon H. O’Neil, Vanessa Schmithorst, Ashok Panigrahy, Rafael Ceschin, Cecilia W. Lo

**Affiliations:** 1 Department of Biomedical Informatics, University of Pittsburgh School of Medicine, Pittsburgh, PA 15206, USA; 2 Division of Cardiology, Department of Pediatrics, Children’s Hospital Los Angeles, Los Angeles, CA 90027, USA; 3 Keck School of Medicine, University of Southern California, Los Angeles, CA 90033, USA; 4 Department of Developmental Biology, University of Pittsburgh School of Medicine, Pittsburgh, PA 15224, USA; 5 Department of Bioengineering, University of Pittsburgh, Pittsburgh, PA 15261, USA; 6 Department of Radiology, University of Pittsburgh School of Medicine, Pittsburgh, PA 15213, USA; 7 Division of Neonatology, Department of Pediatrics, Children’s Hospital Los Angeles, Los Angeles, CA 90027, USA; 8 Department of Pediatric Radiology, UPMC Children’s Hospital of Pittsburgh, Pittsburgh, PA 15224, USA; 9 Department of Anesthesiology Critical Care Medicine, Children’s Hospital Los Angeles, Los Angeles, CA 90027, USA; 10 Department of Critical Care Medicine, University of Pittsburgh, Pittsburgh, PA 51213, USA; 11 Division of Cardiology, Department of Pediatrics, UPMC Children’s Hospital of Pittsburgh, Pittsburgh, PA 15224, USA; 12 Cardiac Neurodevelopmental Care Program, UPMC Children’s Hospital of Pittsburgh, Pittsburgh, PA 15224, USA; 13 Division of Neurology and Child Development, Department of Pediatrics, UPMC Children’s Hospital of Pittsburgh, Pittsburgh, PA 15224, USA; 14 Division of Neurology, Department of Pediatrics, Children’s Hospital Los Angeles, Los Angeles, CA 90027, USA

**Keywords:** olfactory bulb, hippocampus, cerebellum, feeding outcome, neurodevelopmental outcome, *Ohia* HLHS mouse mutants

## Abstract

**Background:** Brain magnetic resonance imaging (MRI) of infants with congenital heart disease (CHD) shows brain immaturity assessed via a cortical-based semi-quantitative score. Our primary aim was to develop an infant paralimbic-related subcortical-based semi-quantitative dysmaturation score, termed brain dysplasia score (BDS), to detect abnormalities in CHD infants compared to healthy controls and secondarily to predict clinical outcomes. We also validated our BDS in a preclinical mouse model of hypoplastic left heart syndrome. **Methods:** A paralimbic-related subcortical BDS, derived from structural MRIs of infants with CHD, was compared to healthy controls and correlated with clinical risk factors, regional cerebral volumes, feeding, and 18-month neurodevelopmental outcomes. The BDS was validated in a known CHD mouse model named *Ohia* with two disease-causing genes, *Sap130* and *Pchda9*. To relate clinical findings, RNA-Seq was completed on *Ohia* animals. **Findings:** BDS showed high incidence of paralimbic-related subcortical abnormalities (including olfactory, cerebellar, and hippocampal abnormalities) in CHD infants (*n* = 215) compared to healthy controls (*n* = 92). BDS correlated with reduced cortical maturation, developmental delay, poor language and feeding outcomes, and increased length of stay. *Ohia* animals (*n* = 63) showed similar BDS findings, and RNA-Seq analysis showed altered neurodevelopmental and feeding pathways. *Sap130* mutants correlated with a more severe BDS, whereas *Pcdha9* correlated with a milder phenotype. **Conclusions:** Our BDS is sensitive to dysmaturational differences between CHD and healthy controls and predictive of poor outcomes. A similar spectrum of paralimbic and subcortical abnormalities exists between human and *Ohia* mutants, suggesting a common genetic mechanistic etiology.

## 1. Research in Context

### 1.1. Prior Evidence

The number of clinical and research magnetic resonance imaging (MRI) studies in neonatal/infant congenital heart disease (CHD) subjects has increased dramatically in the last two decades. Previous studies have utilized brain MRI scores that have focused on cortical structural maturation and acquired brain injury. Paralimbic-related subcortical regions are important for the development of cognitive and visuomotor functions in early development. Levering a large infant brain MRI dataset and a large-scale genetic mouse screen, we theorized that a paralimbic-related subcortical brain MRI score could assist clinicians with outcome prediction in CHD infants.

### 1.2. Added Value

This work aims to validate a subcortical morphological scoring system that could be applied to either clinical or research brain MRI scans of infants with CHD. Our study primarily demonstrated a high incidence of paralimbic-related subcortical structural abnormalities detected by our brain MRI score compared to controls. Our study secondarily demonstrated that our brain MRI score predicted poor language outcomes, poor feeding outcomes, and increased post-surgical length of stay. We also found that the genetic preclinical model of hypoplastic left heart syndrome, the most severe form of CHD, also demonstrated a similar pattern of paralimbic-related subcortical brain abnormalities.

### 1.3. Implications

This novel scoring system developed by our group has implications for early detection of at-risk CHD individuals for poor outcomes, both neurodevelopmental and quality of life. This subcortical paralimbic brain dysplasia score is a simple tool that can be easily added to neuroradiological workflows that can lead to better outcome prediction for children with CHD. Our scoring system helps us to better serve our population, allowing clinicians and researchers to prognosticate highest risk individuals who will benefit from the earliest forms of intervention.

## 2. Introduction

Congenital heart disease (CHD) affects 1% of live births each year [1,2]. As the surgical care for CHD improves, there remains a lingering increased risk of poor neurodevelopmental outcomes for patients with CHD across the lifespan. The mechanism for neurodevelopmental disabilities in non-syndromic CHD is unknown and is thought to be related to fetal exposure to reduced substrate delivery/hypoxia or a genetic underpinning. Genetic abnormalities are detected in approximately 50% of children with syndromic CHD and in approximately 10% of children without a recognizable clinical phenotype [3]. Significant overlap is present between deleterious de novo mutations and previously reported mutations associated with neurodevelopmental disorders [4], suggesting that genetic mutations that cause CHD may also be important in the etiology of neurodevelopmental deficits [5,6]. Smaller brain volumes and dysmature brain structures are seen in neonates and fetuses with CHD before cardiac surgery, suggesting that prenatal and pre-operative factors, including poor substrate delivery (hypoxia/ischemia) may play an important role in altering brain development [7,8,9]. Preterm CHD infants also have abnormal brain development [10]. Within our current understanding of neurogenesis, there is little known about the role that deep grey and subcortical regions play in mediating poor neurodevelopmental outcomes in relation to genetic alterations. Recent animal models and correlative neuropathological studies suggest that cortical dysmaturation is linked to white matter abnormalities, including developmental vulnerability of the subplate in CHD [11,12]. Recent neuroimaging studies have documented paralimbic-related subcortical morphological abnormalities in CHD patients across the lifespan, [13,14,15,16,17], but the relationship between paralimbic-related subcortical morphological abnormalities and clinical and neurodevelopmental outcomes in CHD is also unknown [18,19,20,21].

Children with CHD are at a higher risk of developing brain dysmaturation, a generalized term encompassing abnormal and delayed development of brain macro- and microstructure [15,22,23,24,25]. CHD patients are also at risk for acquired brain injury, including infarcts and small vessel disease across their lifespans as detected by conventional neuroimaging studies. Clinical neuroimaging studies are also becoming more common to obtain in patients during the peri-operative period based on recently published guidelines. Most conventional semi-quantitative MRI scoring systems in CHD patients have exclusively focused on either acquired brain injury or cortical maturation assessment, particularly in the neonatal period [26,27,28,29,30,31,32,33]. We recently developed and validated an infant semi-quantitative score that extended beyond cortical maturation and acquired brain injury to include morphological alterations in paralimbic-related subcortical structures (including cerebellum, hippocampus, olfactory bulb abnormalities), cerebrospinal fluid (CSF)-related abnormalities (including increased extra-axial CSF in frontotemporal regions), and the corpus callosum, known as a brain dysplasia score (BDS), informed by preclinical models of CHD, specifically hypoplastic left heart syndrome (HLHS). We focused our analysis on paralimbic-related subcortical structures that are known to undergo neurogenesis in early development and across the lifespan, including the olfactory bulbs, hippocampus, and cerebellum. We have recently described a similar pattern of structural subcortical dysmaturation both in human infants with CHD and genetically relevant ciliary motion dysfunction and in relation to preclinical models of CHD including hypoplastic left heart syndrome (HLHS) [34,35,36,37,38]. We have previously shown that the BDS correlates with abnormal neonatal brain white matter connectivity patterns [39] but have yet to validate this scoring system in a large dataset in relation to clinical and neurodevelopmental outcomes. Here, we used quantitative structural brain magnetic resonance imaging (MRI) in infants with CHD to test the hypothesis that subcortical morphological measurements could be assessed using a semi-quantitative scoring system termed brain dysplasia score (BDS) and that these subcortical structures are potential predictors of not only infant cortical maturation and regional brain volumes but also are predictors of poor clinical and neurodevelopmental outcomes [13,14,15,16,22,40,41].

To further validate our subcortical BDS from a genetic perspective, we leveraged the *Ohia* mouse model of HLHS recovered from a large-scale mouse mutagenesis screen [42]. In *Ohia* mice with HLHS, phenotypic mutations primarily arise from homozygous mutations in two genes: a chromatin-modifying protein Sin3A-associated protein 130 (*Sap130*) and protocadherin A9 (*Pcdha9*), a cell adhesion protein in the a-protocadherin gene cluster [43]. Importantly, both genes are known to have important roles in cardiac and brain health-related human outcomes [44]. For example, the clustered protocadherins provide cell surface diversity by encoding unique neuronal identity essential for patterning synaptic connectivity [45]. Mice with PCDHA mutations have deficiencies in both brain connectivity and neurobehavioral deficits [46]. Moreover, mutations in both PCDHA and SIN3A are associated with autism and Rett Syndrome in humans [47,48,49]. Importantly, the SIN3A complex also contains transcription factors known to regulate developmental and lifespan neurogenesis [50,51]. Given the prominent roles of PCDHA and SIN3A in human neurogenesis and brain development, we used the *Ohia* mouse model for further validation of the hypothesis that the subcortical BDS could be used as a proxy of subcortical morphological and connectivity quantitative measurements and predict clinical and neurodevelopmental outcomes in human CHD infants.

Our primary aim was to develop an infant paralimbic-related subcortical-based semi-quantitative dysmaturation score, termed brain dysplasia score (BDS), to detect abnormalities in CHD infants (term and preterm) compared to healthy controls and secondarily predict clinical outcomes. Of note, we were interested in correlating gestational age with our BDS and, hence, recruited a preterm CHD group to allow for a large range of gestational ages to be evaluated. Lastly, we also validated our BDS in a preclinical mouse model of hypoplastic left heart syndrome.

## 3. Methods

### 3.1. Ethics

This study was HIPAA-compliant and approved by the institutional review board (IRB) of Children’s Hospital Los Angeles (CHLA) and the University of Pittsburgh Medical Center Children’s Hospital of Pittsburgh (CHP), and written informed consent was obtained from each subject, except for a few patients for which MRI was obtained clinically, and retrospective use of the data was approved by the IRB protocol (CHLA-CCI-09-00055, CCI-10-00235, CHP- 20030213,19100215, 19030204). All experiments were performed per the institutional guidelines and regulations.

### 3.2. Patient Recruitment and Clinical Data Collection

This study is a secondary analysis of neonates with CHD (term and preterm) undergoing brain MRI scans who were recruited as part of a prospective, observational study from two large medical centers, CHLA and CHP. Neonatal CHD cases were prospectively recruited from (1) pregnancies with fetal CHD confirmed with fetal echocardiography, (2) postnatal admissions to the cardiothoracic intensive care unit postoperatively between 2002–2016, and (3) infants with both preterm and term CHD undergoing clinically indicated brain MRI scans at approximately term-equivalent gestational age (GA) were recruited both prospectively and retrospectively in the peri-operative period from 2002–2017. The inclusion criterion was critical CHD defined as a heart defect expected to require corrective or palliative cardiac surgery during infancy. Exclusion criteria for the CHD included documented major chromosomal abnormalities and major congenital brain malformations. Normal referents were recruited (1) from healthy pregnant volunteers and (2) postnatally from a normal newborn nursery. An additional comparison group was included, which was comprised of preterm infants without CHD who were recruited from a high-risk NICU at the same institutions as previously published [21,52,53,54].

**Figure 1:**
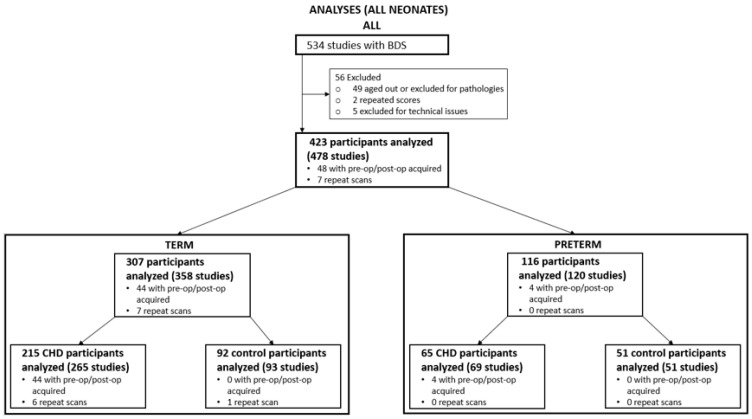
Recruitment Flowchart and Participants Numbers. Study initially consisted of 534 participants, which were scored using the brain dysplasia score (BDS). Participants included in the analysis consisted of 307 term and 116 pre-term individuals.

There were 423 participants who met the inclusion criteria for this study: 307 full-term neonates (215 with CHD and 92 term controls) and 116 preterm neonates (65 with CHD born 25–36 weeks GA and 51 preterm control patients born 24–36 weeks GA) (Figure 1). This is the dataset for which we performed the primary analysis of calculating a BDS and comparing the difference between groups.

### 3.3. Research Scans

Brain MRIs for CHD infants were obtained in either the preoperative (usually between 1–7 days) or postoperative period (up to 13 weeks postnatally, but often sooner). The age range for healthy controls ranged between birth and thirteen weeks postnatally, encompassing the same range of the CHD infants. Preoperative research brain imaging was conducted on CHD subjects when the cardiothoracic intensive care unit team/cardiology team determined the patient was stable for transport to the MRI scanner. Postoperative research scans were performed at less than three months of postnatal age either as an inpatient or outpatient. Most of our scans were research indicated, and as such no additional sedation/anesthesia was given for the scan purpose. Most of the pre-operative scans were performed on non-intubated non-sedated patients; however, if the patient was intubated and sedated for clinical reasons at the time of the scan, their clinically indicated sedation continued under care of the primary cardiothoracic intensive care team. The post-operative scans were performed when the infant was clinically stable and thus were performed as “feed and bundle” scans without sedation. To minimize movement during imaging, infants were secured in Med-Vac Immobilization Bags (CFI Medical, Fenton, MI, USA) with multiple levels of ear protection, including ear plugs, MiniMuffs (Natus Medical Incorporated, Middleton, WI, USA), and standard headphones.

### 3.4. Clinical Scans

Patients in the clinically indicated brain MRI group were scanned at approximately term-equivalent GA either in the pre- or post-operative period as previously published [55]. The post-operative scan for both preterm CHD and term CHD groups was performed at least within 52 weeks corrected postconceptional age.

### 3.5. Neonatal Brain MRI Protocol

MRI studies were acquired: (1) GE 1.5T (Signa LX, GE Healthcare, Milwaukee, WI, USA) MR system using a custom-built neonatal transmit-receive head coil, (2) Philips 3T Achieva MR System (Ver. 3.2.1.1) using standard 8-channel SENSE head coil; and (3) Siemens Skyra 3T scanner using a 20-channel coil. Conventional imaging studies were acquired with the MRS studies and included a 3D coronal SPGR sequence (TE = 6 ms; TR = 25 ms, FOV = 18 cm; matrix = 256 × 160; slice thickness 1.5 mm, spacing 0 mm) or axial and sagittal T1-weighted FLAIR sequences (TE = 7.4, TR = 2100; TI = 750; FOV = 20 cm; Matrix = 256 × 160), axial T2-weighted FSE sequence (TE = 85 ms, TR = 5000 ms, FOV = 20 cm, matrix = 320 × 160 or 256 × 128), and a diffusion-weighted sequence (TE = 80; TR = 10,000; FOV = 22 cm; Matrix = 128 × 128; slice thickness = 4.5 mm, spacing 0 mm). The 3D T1-weighted, T2-weighted, and diffusion-weighted images were reviewed by two pediatric neuroradiologists for evidence of punctate white matter lesion, hypoxic-ischemic injury, acute focal infarction, and hemorrhage as previously described and were used to construct dichotomized and composite brain injury scores [56].

### 3.6. Subcortical Brain Dysplasia MRI Score (BDS) Derivation

Our development of the subcortical-based BDS has been derived from observations from both preclinical mouse CHD mutants and human CHD infants from recent studies from our group [22,23,34,35,36,37,57]. The evaluation for brain dysplasia in our human CHD population is conducted by examining the following structures: cerebellar hemispheres (both left and right together), cerebellar vermis, right olfactory bulb, right olfactory sulcus, left olfactory bulb, left olfactory sulcus, hippocampus, choroid plexus, brain stem, corpus callosum, and supratentorial extra-axial fluid. Supplemental Figure S1 depicts a visualization of how the BDS was calculated. Except for the supratentorial extra-axial fluid, each of the structures listed were evaluated on whether they appear normal or abnormal. For cerebellar hemispheres and cerebellar vermis, they were scored for hypoplasia (small volume) and dysplasia (abnormal shape). Likewise, the olfactory bulbs and sulci were evaluated as separate structures for the purposes of scoring. Based on the finding, a binary number score is given for each structure, with a score of 0 assigned for normal and a score of 1 for abnormal. For olfactory bulbs and sulci, both hypoplastic and absent findings are treated as abnormal and given a score of 1. For supratentorial extra-axial fluid, the structural features demonstrate different gradations of abnormality—none, mild, moderate, or severe fluid accumulation—and thus successively higher integer scores are assigned as the severity of abnormality becomes greater. The integer scores for each of the structures were then summed to create the BDS with “olfactory correction”, which treats both absent and hypoplastic olfactory abnormalities as one score point, thus making the weighting of the olfactory system in line with cerebellum and hippocampus. Higher BDS values can be thought of as having a “worse” score as subjects had more abnormalities present. A subset of cases was reviewed by two raters to validate the scoring.

Similar to our BDS, a brain injury score was calculated for each human participant. The composite brain injury score consisted of four criteria with each criterion being scored as a 1 if present in the participants. The four criteria were hemorrhage, infarct, hypoxic ischemic injury, and periventricular white matter injury. A dichotomized version was also calculated for each individual and was scored as a 1 or 0. If the original brain injury score was greater than 0, then the composite score was a 1; otherwise, the score was a 0. A visual depiction of the brain injury scores can be found in Supplemental Figure S2.

### 3.7. Structural Co-Variate Analysis: Cortical Maturation Score and Regional Brain Volumetric Analysis

We correlated BDS with a classic cortical Total Maturation Score (TMS) and regional brain volumes. Cortical TMS was calculated for all participants with brain MRI included in this analysis. A subset of the full cohort (term controls and CHD scanned at 3T imaging, *n* = 105) underwent cerebral regional volumetric segmentation for structural co-variate analysis. To determine the relationship between our qualitative BDS and quantitative regional morphological dysmaturation, we performed regional brain morphometric techniques including volumetric segmentation of total intracranial cerebral spinal fluid (CSF), cortical grey matter, cortical white matter, deep grey nuclei, brainstem, and cerebellum using a neonatal and infant brain segmentation age-specific atlas. We further segmented total intracranial CSF into three compartments: supratentorial extra-axial CSF, infratentorial extra-axial CSF, and intraventricular CSF. Our group used the above age-specific atlas to build a semi-automated brain parcellation pipeline for use in neonates and young infants (Supplemental Figure S3). The automated processing pipeline, which was previously described by our group [23], was developed using Nipype and interfaces with several image registration algorithms using age-appropriate neonatal/infant templates to accommodate different post-conceptional ages in the patient cohort. Our methodology for volumetric segmentation of neonatal brains has been previously described [58,59]. We further refined this methodology for the current study because of the wide range of post-conceptional ages of neuroimaging studies and the resulting need to use a range of neonatal and infant age-appropriate templates. We also performed validation experiments to ensure optimal performance with this neonatal/infant CHD cohort with mild brain dysplasia. We processed each patient’s T1 and T2 volumetric images in parallel to optimize registration parameters uniquely to each tissue contrast. First, the images were cropped, and the brain was extracted using FSL BET. The volumetric images (T1 and T2 separately) were then bias field corrected using FSL’s FAST segmentation. We then registered the bias-field corrected images to the tissue contrast specific and post-conceptional age-matched template, created by Serag et al. [60], using a non-linear registration from Advanced Normalization Tools (ANTs). The output transformations were inversed and applied to the template space tissue probability maps provided with the neonatal atlas. This transformation results in subject–space tissue probability maps. We calculated tissue volumes by thresholding the partial volume maps at a visually acceptable lower bound and extracting the volume from the binarized mask. The subject–space segmentations were manually checked for accuracy by a pediatric neuroradiologist (A.P.) blinded to patient diagnosis.

### 3.8. Clinical Risk Factor Analysis

We correlated BDS with clinical risk factors. Analysis of clinical risk factors included a subset of participants with critical CHD who required corrective or palliative cardiac surgery within the first month of life who were prospectively enrolled for pre- and postoperative brain MRI scans from 2009–2016. Two hundred ninety-one participants were prospectively enrolled from June 2009 to October 2016. Of these participants, 158 met the exclusion criteria including 57 with no MRI performed, 38 due to prematurity, 38 passed the age threshold, 11 expired preoperatively, 10 had no neonatal surgery, and 4 had a postnatal major genetic diagnosis. Of the 133 term CHD infants with brain MRI meeting the inclusion criteria, 90 participants had sufficient imaging quality for structural analysis and clinical risk factor data collected comprised the study group for this analysis. Participants that had a known major chromosomal abnormality, were premature (<=36 weeks of age), died prior to MRI, or did not require neonatal cardiac surgery were excluded in this analysis. Clinical data were collected from the electronic medical records and included 18 patient-specific and 9 preoperative variables associated with preoperative scan and 6 intra-operative (e.g., cardiopulmonary bypass, deep hypothermic circulatory arrest times) and 12 postoperative variables associated with postoperative scan, as described previously [39,61]. CHD lesions were classified in several ways (not mutually exclusive), including postnatal cyanosis, presence of aortic arch obstruction, single vs. double ventricles, d-transposition of the great arteries, conotruncal defects, heterotaxy, whether the lesion alters fetal cerebral substrate delivery, and severity of this alteration (normal, altered, severely altered).

### 3.9. Feeding Outcomes

We correlated the BDS with feeding outcomes. The feeding outcome study group consisted of term infants with CHD who had neonatal brain MRI with brain dysplasia score between 2003–2015, enrolled both prospectively for research MRI and retrospectively after clinical MRI, at CHLA and CHP. Participants with major gastrointestinal anomalies or surgeries, a diagnosis of CHARGE syndrome, death within the first 30 days of life, or death prior to initiation of feeds were excluded from the feeding analysis. For the feeding study, we evaluated 177 term infants with CHD who had MRI that were used to calculate BDS as described above. Of those, 32 were excluded, including 14 with major gastrointestinal anomalies or surgeries, 1 with a brain anomaly, 6 with CHARGE syndrome, and 11 that died in the first 30 days or prior to initiation of feeds. Therefore, the feeding analysis group consisted of 145 subjects, of which 81 were from CHLA and 64 from CHP. The inpatient medical records were assessed for the following feeding-related variables: presence of dysphagia, aspiration, gastroesophageal reflux, gastrointestinal dysmotility, intestinal malrotation, vocal cord paralysis, use of enteral tube feeding (including nasogastric, nasojejunal, or surgically placed gastric or jejunal tubes), lack of oral feeding prior to neonatal hospital discharge, and length of neonatal hospitalization.

### 3.10. Neurodevelopmental Outcomes

For neurodevelopmental outcomes assessment, Bayley Scales of Infant (version III) and Toddler Development or Battelle Developmental Inventory (version III) were completed by a licensed psychologist in a subset of participants. A total of 90 subjects (CHLA *n* = 32, CHP *n* = 58) had early neurodevelopmental outcome data obtained between 15 to 18 months postnatal age. The standard score for each developmental domain (motor, language/communication, cognitive, and social/emotional) was evaluated. Developmental delay was defined as a standard score greater than 2 standard deviations below the mean in 1 developmental domain. Global developmental delay was defined as a standard score greater than 2 standard deviations below the mean in 2 or more developmental domains. The following criteria were used: Average = Standard Score 90 to 109 (25th to 74th percentile); Low Average = Standard Score 80 to 89 (9th to 24th percentile); Below Average = (2nd to 8th percentile); Exceptionally low = (<2nd percentile). The data were then dichotomized to yes/no for (1) developmental delay, (2) global developmental delay, and (3) below average for each subject to facilitate harmonization between the two different test exams across both sites.

### 3.11. Mouse Screen Analysis

A retrospective analysis was carried out on the mouse lines described by Li et al. [62]. In brief, a forward recessive mouse screen was carried out using N-ethyl-N-Nitrosourea in which 3700 mouse lines, defined by G1 sire, were screened for defects. Defect screening was completed through ultrasound scanning, micro-computed tomography (CT), micro-magnetic resonance imaging (MRI), and visual inspection of G3 offspring. Resulting mutant cardiac phenotypes were confirmed through necropsy or histopathologic analysis. Further analysis was completed by examining the craniofacial and gross brain abnormalities discovered within the screen. Lines in which a mutant was recovered in the original screen were analyzed for both craniofacial and gross brain abnormality. Consistent phenotype in at least three mice was required for a line to be considered abnormal with a craniofacial or brain abnormality. An additional mouse model that recapitulates the phenotype of Joubert syndrome was analyzed prior to any analysis on the main *Ohia* line to validate method and findings. The mouse model was previously validated by Damerla et al. [63]. This Joubert syndrome mouse model is colloquially called Heart Under Glass (Hug) and contains an S235P missense mutation in Jbts17. The Hug mice recapitulate the brain phenotype of Joubert syndrome presenting with decreased number of cerebellar fissures.

### 3.12. Episcopic Confocal Microscopy (ECM)

Head samples were removed following necropsy and fixed in 4% PFA solution for a minimum of 72 h. After fixation, samples were prepared for ECM via three graded ethanol baths of 10%, 20%, and 40% overnight at room temperature. Samples were then transferred to a Sakura Tissue Tek VIP 5 Tissue Processor where they were dehydrated using increasing percentages of warmed ethanol baths and eventually perfused with paraffin wax. Samples were embedded in paraffin blocks to be sectioned coronally. Three-dimensional reconstructed image stacks were used for brain scoring and volumetric analysis. A diagram for the ECM workflow can be found in Supplemental Figure S4.

### 3.13. Mouse Brain Dysplasia Scoring

A group of three reviewers reviewed 84 cases consisting of 69 *Ohia*, 5 CRISPR/CAS9, and 10 wild type samples. Reviewers came to a consensus for each structure scored. In total, seven structures were included in the mouse brain scoring, including the hippocampus, cerebellum, cerebrum, left and right olfactory bulbs, brain stem, and midbrain. Hippocampus, cerebellum, and both olfactory bulbs were scored for aplasia, hypoplasia, and dysplasia. Cerebrum, brain stem, and midbrain were scored for hypoplasia and dysplasia only. Each instance of abnormality received a binary score of zero or one. Brain Dysplasia Score (BDS) was a sum of each area of the brain. Two other BDS metrics were calculated, one being a binary scoring of any cerebellar or hippocampal abnormalities and the other being a binary score if any abnormality was present in the sample at all. Cerebellar folds were counted using a sagittal view in the middle slice of the brain.

### 3.14. Mouse Brain Segmentation

Tiff images were converted to NIFTI images using ITK-Snap and FSL. Images were down sampled 4x using FMIRB’s Linear Registration Tool (FLIRT). The resulting images were manually segmented into 16 discreet structures using ITK-Snap. Structures segmented included the right hippocampus, right olfactory bulb, right subcortical area, right cortex, intracranial space, midbrain, left hippocampus, left olfactory bulb, left subcortical area, left cortex, extra-axial space, cerebellum, pons, medulla, hypothalamus, and choroid plexus. Due to the required processing methods for ECM, CSF is removed from the brain and replaced with paraffin wax. The space voided of CSF has been labeled as intracranial space and extra-axial space. Structures above the tentorium were combined to form a supratentorial volume, and an infratentorial volume was also calculated. Infratentorial volumes consisted of the pons, medulla, and cerebellum, while the supratentorial volumes were composed of the hippocampus, hypothalamus, olfactory bulbs, subcortical areas, cortex, and midbrain.

### 3.15. Genotype Analysis

We explored the relationship of Sap130/Pcdha9 genotype and brain morphology in the mouse modeling. When comparing the risk stemming from the BDS for animals from the *Ohia* line, different genotypes were grouped into 6 distinct groups. Group A: *Sap130* (m/m) and *Pcdha9* (m/m), Group B: *Sap130* (m/m) and *Pcdha9* (m/+), Group C: *Sap130* (m/m) and *Pcdha9* (+/+), Group D: *Sap130* (m/+) and *Pcdha9* (m/m), Group E: *Sap130* (+/+) and *Pcdha9* (m/m), and lastly, Group F: *Sap130* (m/+) or (+/+) and *Pcdha9* (m/+) or (+/+).

### 3.16. Mouse Genotype Groupings and Comparisons

For greater power in subsequent analysis, mice with similar genotypes were pooled into Groups. Group ABC consisted of mice that were homozygous mutant for *Sap130* (m/m), irrespective of their *Pcdha9* genotype. An additional Group, AB, contained mice that were homozygous mutant for *Sap130* (m/m), and either homozygous mutant or heterozygous for *Pcdha9* (m/*). To study the effect of homozygous mutations in the *Sap130* gene, Group ABC was compared against wildtype animals. Group ABC was then compared against Group F. Group F can only be heterozygous for *Sap130* and *Pcdha9*, so any effect of the homozygous mutant *Sap130* alleles would be removed in this group. This pairing should show effects of the homozygous *Sap130* mutation more concretely.

To assess if any of the effect is coming from the *Pcdha9* genotype, Group AB was then compared to F. Group AB differs from Group ABC by removing samples that contained wildtype *Pcdha9* genotyped animals. If *Pcdha9* is partially, or in large part, responsible for the observed phenotype, removing wildtype *Pcdha9* samples from the ABC Group should increase incidence and effects should become more significant. To further isolate the effect of *Sap130*, Group A was compared against Group C with the difference between groups being homozygous mutant *Pcdha9* (Group A) vs. wildtype (Group C). Lastly, Group B was compared against Group D. Group B is homozygous *Sap130* and heterozygous *Pcdha9*, and Group D is the opposite, *Sap130* heterozygous, and *Pcdha9* homozygous mutant. If either homozygous gene is more significant than the other, the effect could be seen within this comparison.

### 3.17. RNAseq Analysis

RNA was isolated from brain tissue samples of 4 *Ohia* mutant animals and 5 littermate controls at embryonic day E13.5-E14.5, libraries were constructed and sequenced on the Illumina HiSeq 2000 platform (BGI Americas, Cambridge, MA, USA), and differential gene expression analysis was conducted as previously described [64]. The online Database for Annotation, Visualization, and Integrated Discovery (DAVID) was used to perform gene ontology functional enrichment analysis.

### 3.18. Statistical Analyses for Human Studies

Primary analysis: SAS statistical software was used to carry out human statistical analysis. Our primary analysis compared the derived BDS between neonates with CHD and controls. This analysis was performed using multivariate regression and three major co-variates were included in the model: gender, postconceptional age (PCA = gestational age plus postnatal age), time of MRI scan timing of scan relative to cardiac surgery (pre- vs. post-operative). Multi-variate regression with false discovery rate (FDR) correction was used, to correct for multiple comparisons. The FDR is one way of conceptualizing the rate of type 1 errors in null hypothesis testing when conducting multiple comparisons. Whether BDS is association with sex difference, GA, or PCA was assessed in the entire study cohort.

Secondary analysis: Secondary analysis was repeated within the CHD only group, with birthweight and the presence of genetic abnormality included as additional comparisons with BDS. Furthermore, whether pre- or post-operative and pre-term or term status correlated with BDS was also analyzed within the CHD group. As an additional secondary analysis, within the CHD group, BDS was compared to the presence of various injuries and cortical maturation features. Additional secondary analyses including correlation of BDS with feeding data and neurodevelopmental outcome, which consisted of linear and logistic regression with false discovery rate (FDR) correction for multiple comparisons; FDR-adjusted *p*-value < 0.05 was considered significant.

### 3.19. Statistical Analyses for Mouse Studies

Volumetric analysis of mouse individual structures was carried out using SAS statistical software (Cary, NC, USA). Both raw and normalized by total brain volume values were compared in any models generated. We completed an ad hoc analysis to delineate the relationship between BDS and regional brain volumes, clinical risk factor and heart lesion subtypes on our primary outcome measures. Clinical variables were compared using a three-way ANCOVA (analysis of covariance) between wild-type, *Ohia*, and Hug animals. We then compared each of the groups mentioned above to each other using pairwise *t*-tests.

## 4. Results

### 4.1. Comparison of Brain Injury/Cortical Maturation Score (TMS) between CHD and Control Cohorts

The preterm CHD and term CHD cohort demonstrated increased incidence of brain injury compared to their gestational-aged, matched controls. Specifically, the CHD preterm cohort demonstrated increased incidence of focal infarcts (*p* = 0.0295) and punctate white matter lesions (*p* = 0.0231) compared to the preterm control cohort. The term CHD cohort demonstrated increased rates of hemorrhage (*p* = 0.0284), focal infarct (*p* = 0.0035), punctate white matter lesions (*p* < 0.001), dichotomized injury composite score (*p* < 0.001), and injury composite score (*p* < 0.001) compared to the term control cohort (Supplemental Table S1).

When comparing the categories of the cortical maturation score (TMS), the term CHD cohort had widespread reduced cortical maturation (in all cortical lobes except occipital) compared to term control cohort, while the preterm CHD only differed from preterm controls in the myelination category (Supplemental Table S2).

### 4.2. Derivation of Human BDS in Human Infant CHD

There was a high incidence of olfactory, hippocampal, and cerebellar abnormalities in the preterm and term CHD cohorts compared to both preterm and term control cohorts respectively (Table 1). Within the univariate analyses, there was no significant correlation between BDS and sex, GA, or PCA within the entire study cohort (Table 2). Within the CHD cohort, there was no correlation between BDS and pre/postoperative status, preterm/term status, or birth weight. Higher BDS in the CHD cohort was correlated with known genetic abnormalities (Table 3), which remained a significant association in the multivariable analysis (Table 4). BDS was highly correlated with cortical immaturity by cortical maturation scores including frontal cortex (*p* < 0.0001), insular cortex (*p* < 0.0001), and cortical folding (*p* = 0.0016), even after controlling for PCA at the scan (Table 5). There was no significant correlation between BDS and any categories of brain injury (Table 5). Within a subset of cases, the inter-rater reliability was calculated and resulted in reviewers one and two receiving high scores for most metrics included in the BDS calculation. Notable exceptions were for cortical folding (kappa = 0.18, 0.00), myelination (kappa = 0.35, 0.02), supratentorial extra-axial fluid (kappa = 0.35, 0.05), and germinal matrix (kappa = 0.48, 0.00). Other values for Cohen’s kappa measure for the BDS can be found in Supplemental Table S3.

### 4.3. Human Infant BDS and Regional Cerebral Volumes

Higher BDS correlated with smaller left and total cerebellar volume, smaller deep grey matter and brain stem volumes, and increased infra-ventricular and supra-tentorial CSF volumes in the entire sample (term CHD and term control cohorts combined) (Table 6). Increased BDS was more strongly correlated with reduced cerebellar volume, reduced cortical volumes, and increased CSF volume in the term CHD cohort compared to the term control cohort (Table 6).

### 4.4. Human Infant BDS and Clinical Risk Factors

When assessed against clinical risk factors in term CHD infants, the BDS did not correlate with birth factors, anthropomorphic data, cardiac lesion type, or intraoperative factors. (Supplemental Table S4). The BDS did correlate with age of under 7 days at surgery (*p* < 0.048, Supplemental Table S4), increased length of hospitalization (*p* < 0.0447, Supplemental Table S4), and need for G-tube placement during the neonatal hospital stay (*p* < 0.01, Supplemental Table S4). Of note, we found no correlation between BDS and MRI field strength/scanner type (Supplemental Table S5).

### 4.5. Human Infant BDS and Feeding Outcomes

**Figure 2:**
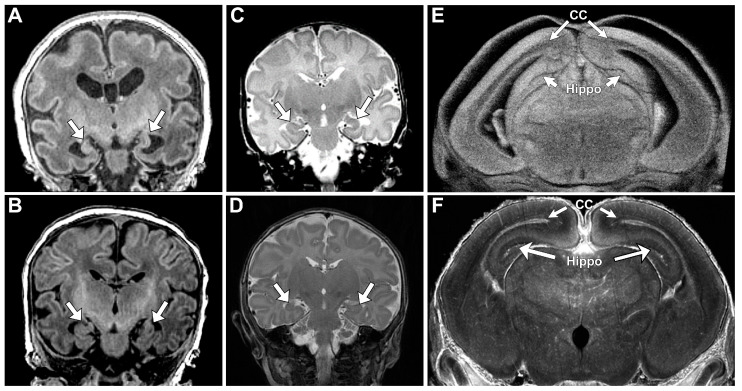
Hippocampal Abnormalities Were Similar Among Human and Mouse. Panels (**A–D**) demonstrate abnormal hypoplastic and/or malrotated hippocampi (marked with arrows) in human infants with CHD. (**E**) demonstrates comparable abnormalities in the mouse mutant with abnormal hippocampus (Hippo) and corpus callosum (CC) marked with arrows. (**F**) Depicts a normal mouse hippocampus (Hippo) and corpus callosum (CC), marked with arrows.

**Figure 3:**
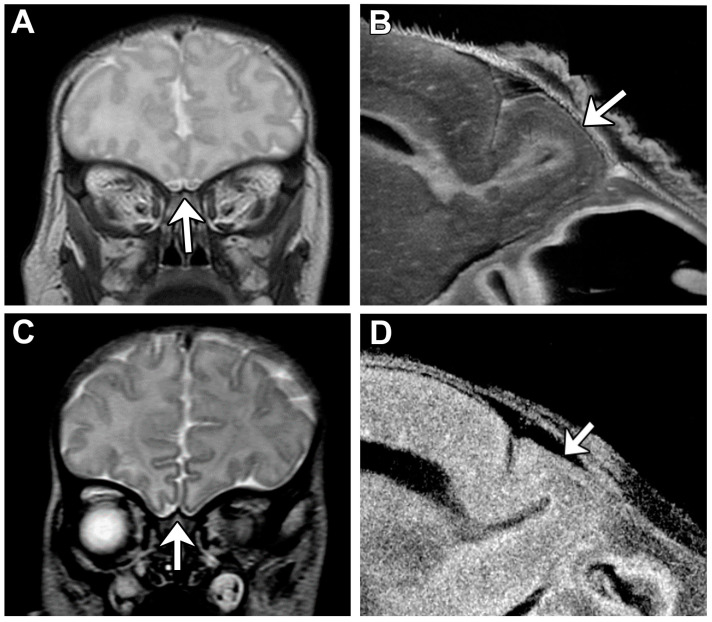
Consistent Olfactory Bulb Phenotype is Visible Among Human and Mouse. Olfactory bulbs are demonstrated with an arrow in each panel. (**A**) Human control subject exhibits normal olfactory bulb size and structure. (**B**) Mouse control showing olfactory bulb with normal shape and size. (**C**) HLHS human subject with aplastic olfactory bulbs. (**D**) Mouse with congenital heart disease with hypoplastic olfactory bulb.

**Figure 4:**
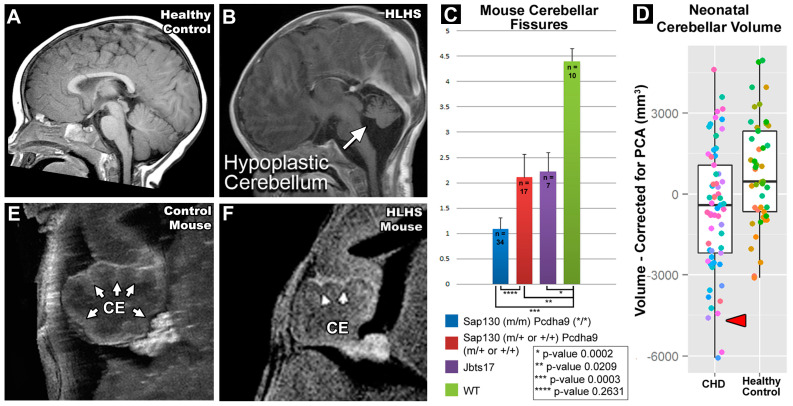
Cerebellar Abnormalities Seen in Both Human and Mouse. Panels (**A**,**B**) depict a human subject with HLHS compared to a healthy control. The cerebellum of the HLHS subject is hypoplastic and demonstrates dysmaturation. (**C**) Average number of cerebellar fissures among mouse groupings. (**D**) Human neonatal cerebellar residual volumes with gestational age regressed out. Panels (**E**,**F**) show the cerebellum (CE) of a mouse with HLHS and a normal control. The cerebellum of the HLHS mutant displays hypoplasia and a lesser number of cerebellar fissures (marked with arrows).

Lack of oral feeding before neonatal hospital discharge was associated with multiple morphological abnormalities of the components of the BDS including cerebellar hemispheres and vermis, hippocampus, bilateral olfactory bulbs and olfactory sulci, corpus callosum, increased supratentorial extra-axial fluid, as well as total increased BDS (Table 7). Hospital length of stay was associated with dysplasia of the cerebellar hemispheres and vermis, hippocampus, choroid plexus, brainstem, increased supratentorial axial fluid, and BDS (Table 7). A diagnosis of dysphasia was associated with dysplasia of the brain stem ((*p* = 0.001) Table 7). Gastrointestinal dysmotility, aspiration, gastroesophageal reflux, malrotation, and vocal cord paralysis demonstrated no significant associations with brain dysplasia (Table 7). Figure 2 panels A–D show some of the variations in hippocampal anatomy seen in the human population compared against that seen in the mouse in panels E and F. Figure 3 demonstrates olfactory bulb abnormalities in human infant CHD vs. control (A/C) and the mouse model (B/D). Figure 4 demonstrates cerebellar abnormalities in the human infant CHD vs. controls (A,B) and the mouse model (E,F).

### 4.6. Human Infant BDS and Neurodevelopmental Outcomes:

Increased BDS correlated with developmental delay in at least one domain including motor, cognitive, language/communication, or social/emotional (*p* < 0.02), and specifically with poor expressive language development (*p* < 0.016) in a subset of the original sample tested at 15–18 months of age (*n* = 90), given in Table 8.

### 4.7. Mouse CHD Screen Results

**Figure 5:**
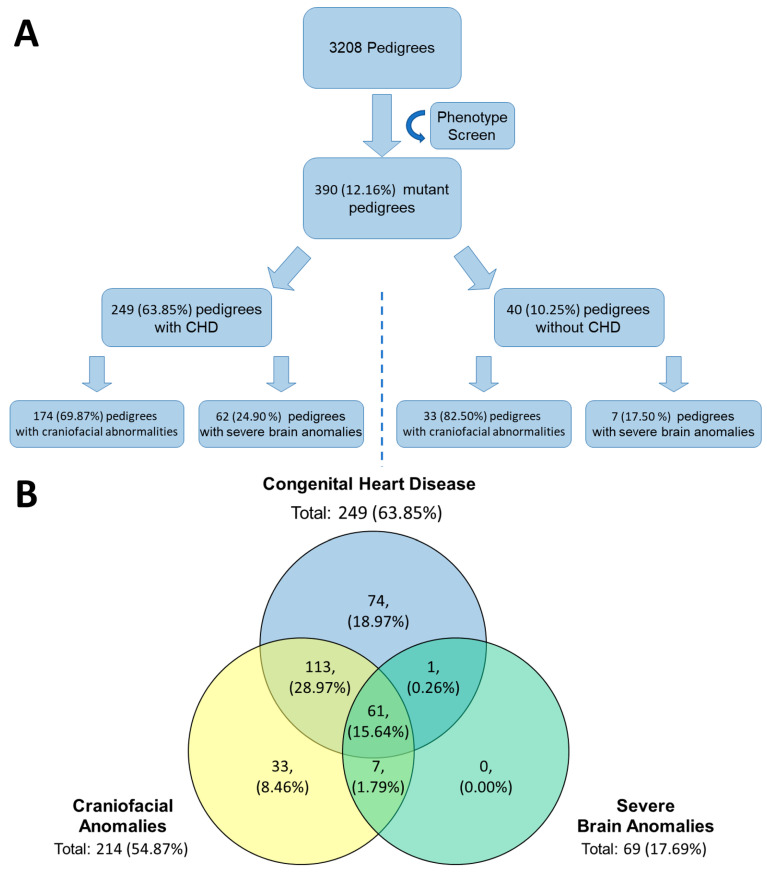
Phenotype Mouse Screen Recovered Mutant Mice with Defects in Cardiac, Brain, and Craniofacial. (**A**) From the original phenotype screen 390 mutant lines were identified. Of those 390 lines, 249 had CHD and of those, 174 and 62 had craniofacial and severe brain anomalies, respectively. Of the 40 lines that did not have CHD, 33 had craniofacial and 7 had severe brain anomalies. (**B**) Three major groupings, congenital heart disease, craniofacial anomalies, and server brain anomalies, identified from the original phenotype screen and their relationship to one another. The highest overlap between two conditions is congenital heart disease and craniofacial anomalies in approximately 29% of the original 390 mutants identified.

To further validate our subcortical BDS from a genetic perspective, we leveraged the *Ohia* mouse model of HLHS recovered from a large-scale mouse mutagenesis screen [42]. In *Ohia* mice with HLHS, phenotypic mutations primarily arise from homozygous mutations in two genes, a chromatin modifying protein Sin3A-associated protein 130 (*Sap130*), and protocadherin A9 (*Pcdha9*), a cell adhesion protein in the a-protocadherin gene cluster [43]. From the original mouse screen for CHD, 3,208 mouse pedigrees were screened, and 390 (12.16%) mutant lines were discovered. Of the 390 mutant lines, 214/390 (54.87%) had some form of craniofacial anomaly and 69/390 (17.69%) lines had a severe brain anomaly. Within those categories, craniofacial and brain anomalies, 174/214 (81.31%) and 62/69 (89.86%) had co-occurrence of CHD, respectively. Supplemental Figure S5 demonstrates some of the craniofacial abnormalities seen during the screening. A total of 68 lines contained features of both types of anomalies, and of those 68 lines, 61 also exhibited CHD. A summary of the results can be seen in Figure 5.

### 4.8. Hug Model Mouse ECM Validation

To validate the ECM method, a mouse model, colloquially called Heart Under Glass or Hug, for Joubert syndrome with a S235P missense mutation in Jbts17 was analyzed prior to analysis of *Ohia* mice. Joubert syndrome is a ciliopathy associated with cerebellar abnormalities and other birth defects. In addition, Hug mice also had higher incidences of BDS (*p* = 0.0161) and specifically in the cerebellum-hippocampus dichotomized BDS (*p* = 0.0154). Compared to wildtype mice, Hug mice showed an overall increase in cerebellar abnormalities (*p* < 0.001) and a decrease in the number of cerebellar fissures (*p* = 0.0023).

### 4.9. Ohia Mouse Model Brain Dysplasia Scoring

ECM analysis was completed on 69 *Ohia* mutant neonatal mice. There was an increase in the BDS in the *Ohia* group compared to the wildtype group (*p* < 0.001) (Table 9). With regards to individual subcortical abnormalities (which make up the composition of the BDS), the cerebellum showed the highest incidence of abnormality with dysplasia being present in 49/67 (73.13%) of animals scored and was highly significant compared to control animals (*p* < 0.001). *Ohia* mutants also showed a decrease in the number of cerebellar fissures (*p* < 0.001) compared to the wild type group. The *Ohia* mutants also showed an increase in incidence of hippocampal dysplasia (*p* = 0.002), cerebral dysplasia (*p* = 0.003), left olfactory bulb aplasia (*p* = 0.024), left olfactory bulb any abnormality (*p* = 0.006), right olfactory bulb aplasia (*p* = 0.02), and right olfactory bulb any abnormality (*p* = 0.007) (Table 9). Supplemental Figure S6 demonstrates some of the common phenotypes seen in *Ohia* mice. Findings of aplasia, hypoplasia, or dysplasia were common in the olfactory bulb, cerebellum, and/or hippocampus. Additionally holoprosencephaly was a coincidental finding in *Ohia* mice, with 42/69 (60.9%) showing some signs of holoprosencephaly. Supplemental Figure S7 shows *Ohia* animals and two examples of the holoprosencephaly observed in this population.

### 4.10. Ohia Brain Dysplasia Scoring within CHD Subgroupings

To examine the relationship between CHD lesions and brain dysmaturation, the co-occurrence of different brain anomalies and any form of CHD was compared. We compared within the *Ohia* cohort looking at BDS and cerebellar fissures and found that the presence of CHD increased the BDS and reduced cerebellar fissures (*p*- < 0.001) (Table 10). Looking at other forms of cardiac sub-lesions, there were no significant differences between BDS and cerebellar folds (Table 10).

In *Ohia* animals, there were 10 brain structures that showed an increased incidence of abnormalities in animals with CHD. Those structures included hippocampal dysplasia (*p* = 0.043), cerebral hypoplasia (*p* = 0.008), cerebral dysplasia (*p* < 0.001), cerebellar dysplasia (*p* < 0.001), left olfactory bulb aplasia (*p* < 0.001), left olfactory bulb any abnormality (*p* < 0.001), right olfactory bulb aplasia (*p* < 0.001), right olfactory bulb any abnormality (*p* < 0.001), brainstem dysplasia (*p* = 0.016), and midbrain dysplasia (*p* = 0.0062) (Supplemental Table S6). Comparing single versus biventricular morphology, defects were more common in the cerebellum in double ventricular mice (*p* < 0.001) (Supplemental Table S6). When comparing conotruncal vs. non-conotruncal defects, mice with conotruncal defects showed an increase in the occurrence of hypoplasia in the cerebrum (*p* = 0.037) (Supplemental Table S6). Within cyanotic vs. acyanotic cardiac defects, mice with acyanotic defects showed increases in the incidence of left olfactory bulb hypoplasia (*p* = 0.018) and any defect in the cerebellum (*p* = 0.037). Lastly, looking at mice with and without arch obstructions, mice with arch obstruction showed an increase in the occurrence of any defect in the cerebellum (*p* = 0.048) (Supplemental Table S6).

### 4.11. Ohia Mouse Model Regional Brain Volumes

*Ohia* mutants demonstrated a significant decrease in raw (non-normalized) volumes of the right subcortical (*p* = 0.044), left subcortical (*p* = 0.041), cerebellar (*p* = 0.005), and supratentorial volume (*p* = 0.037) volumes (non-normalized) compared to wild type control. *Ohia* mutants also demonstrated an increase in the intraventricular volume (*p* = 0.009) compared to wild type controls (Supplemental Table S7).

Normalizing volumes to total brain volume showed *Ohia* animals also exhibited a significant increase in medullary (*p* = 0.024) and intraventricular normalized volume (normalized to total brain volumes) (*p* = 0.015). *Ohia* animals also demonstrated a decrease in normalized supratentorial volumes (*p* = 0.01) compared to wild type controls (Supplemental Table S7).

### 4.12. Ohia Mouse Model Regional Brain Volumes within CHD Subgroupings

There were no differences in raw (non-normalized) volumes between *Ohia* mice with and without CHD. Looking at single versus biventricular morphology, mice with biventricular heart defects showed increases in the volumes of the right subcortical areas (*p* = 0.006), left subcortical area (*p* = 0.036), and the pons (*p* = 0.024) (Supplemental Table S8). Comparing conotruncal against non-conotruncal heart defects, mice with conotruncal defects showed an increase in the right subcortical area (*p* = 0.043) (Supplemental Table S8). Mice with acyanotic defects demonstrated an increase in the volume of the left hippocampus (*p* = 0.044) (Supplemental Table S8). There were no differences in volume noticed between mice with and without arch obstructions.

After normalizing raw volumes to total brain volume, different regions showed significance between groups. Between CHD and non-CHD *Ohia* mice, *Ohia* mice with CHD showed increased volumes in the right cortex (*p* = 0.026), midbrain (*p* = 0.044), and cerebellum (*p* = 0.044). Comparing normalized volumes within single and biventricular groupings found that single ventricular morphology heart defect mice had increased choroid plexus volumes (*p* < 0.001). (Supplemental Table S9). There were no significant findings looking between groups of conotruncal vs. non-conotruncal, cyanotic vs. acyanotic, or arch obstruction vs. no arch obstruction (Supplemental Table S9).

### 4.13. Ohia Mouse Model Brain Dysplasia Scoring within Genotype Subgroupings

Comparing genotypes within the *Ohia* cohort, samples were first grouped based on genotypes. The two groups with the most samples were group A, which consisted of Sap130 and Pcdha9 double mutants, and group F consisting of animals that did not have a Sap130 or Pcdha9 homozygous mutant genotype (Table 11). Both groups showed high incidence rates of olfactory bulb, cerebellar, hippocampal, and brainstem abnormalities (Table 11). Using Group ABC mentioned in the Mouse Genotype Groupings section in the methods, values of hippocampus and cerebellar BDS (*p* < 0.001) and BDS total (all structures) were greater (*p* < 0.001) in Group ABC compared to wildtype (Table 12).

Within the different genotype groups, there were various findings. Groups A and B had 19/24 (79.17%) and 6/8 (75%) of mice exhibiting some form of holoprosencephaly. Additionally, those groups had high rates of CHD and various subtypes of CHD (i.e., conotruncal, single ventricular morphology, cyanotic defects, and arch obstructions) (Supplemental Table S10).

When comparing ABC vs. WT, to determine homozygous Sap130 mutation difference compared to controls, 10 structures studied showed greater incidence values in ABC compared to wildtype. Those structures included dysplastic hippocampus (*p* < 0.001), hypoplastic cerebrum (*p* = 0.40), dysplastic cerebrum (*p* < 0.001), dysplastic cerebellum (*p* < 0.001), aplastic left olfactory bulb (*p* = 0.001), combination of left olfactory bulb (*p* < 0.001), aplastic right olfactory bulb (*p* = 0.001), combination of right olfactory bulb (*p* ≤ 0.001), dysplastic brainstem (*p* = 0.037), and the dichotomized BDS (*p* ≤ 0.001) (Supplemental Table S11).

Comparing groups ABC vs. F to determine homozygous Sap130 mutation difference compared to no homozygous mutation in either gene (at most hetero or WT for either mutation), hippocampal and cerebellar BDS was different between groups with ABC, a higher incidence (*p* = 0.008) (Table 12). Eight structures had significantly increased incidence in group ABC compared to F. Structures included hypoplastic cerebrum (*p* = 0.021), dysplastic cerebrum (*p* = 0.016), dysplastic cerebellum (*p* < 0.001), aplastic left olfactory bulb (*p* = 0.035), combination of left olfactory bulb (*p* = 0.010), aplastic right olfactory bulb (*p* = 0.022), combination of right olfactory bulb (*p* = 0.018), and dichotomized BDS (*p* = 0.021). Additionally, the ABC group had an increased value for the hippocampus and cerebellum BDS (*p* = 0.008) (Supplemental Table S11).

In comparing groups AB vs. F, (homozygous Sap 130 mutation and homo/heteroPCDHA9 vs. no hippocampal and cerebellar BDS was significantly different between groups (*p* = 0.021) (Table 12). Seven structures showed significant differences in the rate of abnormalities; structures included hypoplastic cerebrum (*p* = 0.035), dysplastic cerebrum (*p* = 0.019), dysplastic cerebellum (*p* = 0.001), aplastic left olfactory bulb (*p* = 0.039), combination of left olfactory bulb (*p* = 0.023), aplastic right olfactory bulb (*p* = 0.023), and combination of right olfactory bulb (*p* = 0.023) (Supplemental Table S11). Comparing groups A vs. C, only two structures were found significant. Those structures were the dysplastic brainstem (*p* = 0.028) and dysplastic midbrain. (*p* = 0.042) (Supplemental Table S11). Comparing groups B vs. D, only the aplastic hippocampus (*p* 0.029) and any defect in the hippocampus (*p* = 0.014) were found to be significant (Supplemental Table S11).

### 4.14. Ohia Mouse Model Regional Brain Volumes within Genotype Subgroupings

Looking at volumes for brain regions between genotype groups, in group ABC vs. wild type, six areas were found to be significantly different with mice in group ABC having reduced volumes including the right subcortical area (*p* = 0.006), right cortex (*p* = 0.047), left subcortical area (*p* = 0.007), cerebellum (*p* = 0.003), supratentorial volume (*p* =0.013), and total brain volume (*p* = 0.028) (Supplemental Table S12).

In group ABC vs. F, mice in group ABC showed significant volume reductions in eight structures. Structures included right hippocampus (*p* = 0.041), right subcortical areas (*p* = 0.050), right cortex (*p* = 0.016), left hippocampus (*p* = 0.031), left olfactory bulb (*p* = 0.048), left cortex (*p* = 0.022), supratentorial volume (*p* = 0.018), and total brain volume (*p* = 0.022) (Supplemental Table S12). Similarly comparing groups AB vs. F, mice in group AB showed significant reductions in volumes in eight structures. Differences occurred between right hippocampus (*p* = 0.021), right subcortical areas (*p* = 0.023), right cortex (*p* = 0.029), left hippocampus (*p* = 0.024), left subcortical areas (*p* = 0.038), left cortex (*p* = 0.029), supratentorial volume (*p* = 0.014), and total brain volume (*p* = 0.014) (Supplemental Table S12). Lastly, comparing group A vs. C, animals in group A showed significant reductions in four areas including the right subcortical area (*p* = 0.022), left subcortical area (*p* = 0.034), infratentorial volume (*p* = 0.047), and total brain volume (*p* = 0.049) (Supplemental Table S12).

After normalizing raw volumes to total brain volume, multiple brain regions showed significant differences between groupings. The ABC group demonstrated increased volume of the midbrain (*p* = 0.027) and medulla (*p* = 0.005) and decreased volume of the right olfactory bulb (*p* = 0.037) and supratentorial region (*p* = 0.009) compared to the wild type groups (Supplemental Table S12). The ABC group demonstrated significant increases in volume in three areas including the cerebellum (*p* = 0.010), medulla (*p* = 0.043), and choroid plexus (*p* = 0.012) compared to the F group. Additionally, the right cortex volume was reduced in animals in group ABC compared to F (*p* = 0.028) (Supplemental Table S13).

Comparing groups AB vs. F, mice in group AB demonstrated three structures with significant increases in volume including the cerebellum (*p* = 0.012), pons (*p* = 0.049), and choroid plexus (*p* = 0.029). There were no significant differences between groups A vs. C and B vs. D (Supplemental Table S13).

### 4.15. Mouse CHD/Ohia RNA-Seq

**Figure 6:**
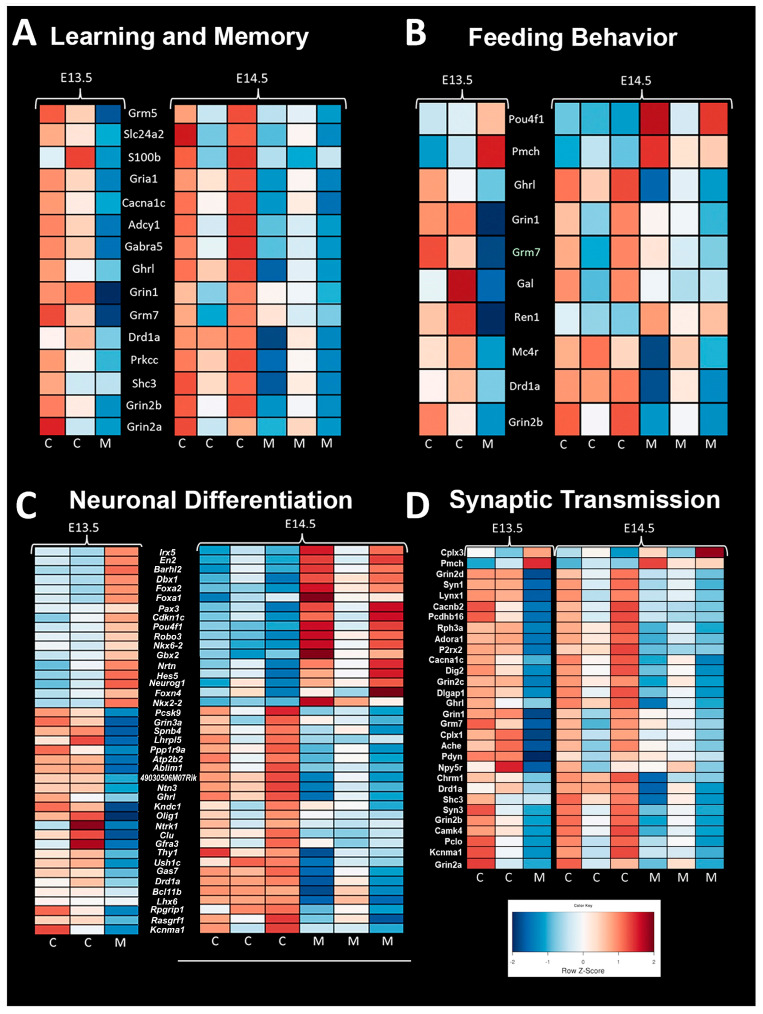
Mouse RNA Sequencing Analysis Shows Significant Difference in Gene Expression. (**A**) Genes associated with learning and memory showed lesser expression in control mice compared to *Ohia* mutant mice. (**B**) Genes associated with feeding behavior showed an abnormal pattern in *Ohia* mice. *Ohia* mice showed a pattern of expression that was opposite compared control mice at both time points. (**C**) Genes associated with neuronal differentiation showed an opposite pattern of expression in *Ohia* mutant mice compared to controls. (**D**) Genes associated with synaptic transmission had a different expression pattern in *Ohia* mutant mice compared to controls.

RNA-seq transcriptome profiling in term *Ohia* brains showed changes in genes involved in learning and memory, feeding behaviors, neuronal differentiation, and synaptic transport (Figure 6). Sap130 chromatin immunoprecipitation sequencing (CHIP-seq) looking at targets associated with Sap130 in brain tissue revealed a total of 644 genes being associated with cardiac- and brain-associated genes. Of those 644 genes, 106 and 324 were cardiac and brain genes, respectively, with the remaining 214 genes being associated with both brain and cardiac. The top three phenotypes associated with the Sap130 CHIP-seq analysis were global brain delay, abnormal brain development, and abnormal nervous system development.

### 4.16. Crisper/CAS Phenotype Validation

Crisper/CAS9 mice were also processed via ECM for validation of phenotypes associated with *Ohia* mice. Three different crisper/CAS mice were developed to validate: a Sap130/Pcdha9 double mutant, Sap130 only, and Pchda9 only. Brain scoring was performed like *Ohia* mice, and findings support a similar phenotype. In the Sap130/Pcdha9 double mutant mice analyzed, they were found to exhibit a severe phenotype characterized by smoothed cerebellum, aplastic or highly dysplastic olfactory bulbs, and an aplastic hippocampus. Other animals showed a pattern of holoprosencephaly. The Sap130 crisper animal showed a severe phenotype as well with smooth cerebellum, absent or extremely abnormal olfactory bulbs, and either holoprosencephaly or an abnormal hippocampus. In the Pcdha9 crisper animal, the phenotype was milder and presented with a cerebellum that, while dysplastic, did present with fissures unlike the double mutant or Sap130 crisper animal, a semi-normal-appearing olfactory bulb, and hippocampus. The phenotype was more severe in animals having mutations in the Sap130 gene. A visual representation of the defects found in Crisper/CAS9 mice can be found in Supplemental Figure S8. A similar pattern of severe olfactory bulb and hippocampal structural abnormalities was seen in our human population. Supplemental Figure S9 shows the Crisper/CAS animals compared to a human subject with severe brain dysmaturation.

## 5. Discussion

Our study demonstrated that a paralimbic-related subcortical-based semi-quantitative BDS has proven to be sensitive to dysmaturational differences between CHD and control infants. We also show secondarily in a subset of these patients that this BDS score can predict neurodevelopmental outcomes within CHD infants, including associations with early language, clinical, and feeding outcomes. Most conventional semi-quantitative MRI scoring systems in CHD patients have exclusively focused on either acquired brain injury or cortical maturation assessment, particularly in the neonatal period [26,27,28,29,30,31,32,33]. In this study, we demonstrated that not only is it possible to generate a paralimbic-related subcortically informed scoring system, but that such a scoring system holds important information regarding outcomes for CHD infants at risk for atypical neurodevelopment. The key subcortical components of our scoring system included subcortical structures that are known to be part of paralimbic neural networks including the olfactory bulb, the hippocampus, the cerebellum, and the deep grey structures like the striatum. Furthermore, we showed that there is a distinct similarity between findings seen in CHD infants when compared to a preclinical mouse model of CHD suggesting similar mechanisms of brain dysmaturation. Interestingly, the subcortical abnormalities identified in our CHD patients (olfactory, cerebellar, and hippocampal abnormalities) have been described in non-CHD pediatric disorders like autism and attention deficit hyperactivity disorder.

In our study, we found a high incidence of paralimbic-related subcortical abnormalities including olfactory, cerebellar, hippocampal, and brainstem abnormalities in infants with CHD compared to controls. We found similar olfactory bulb defects in the preclinical mouse model that we also see in our human CHD population. These abnormalities were used to derive a semi-quantitative subcortical abnormality or brain dysplasia score (BDS). We found that this BDS correlated with reduced cortical maturation (measured with the classic TMS), increased CSF volume and increased deep grey volume (striatum/thalamus). In contrast, the BDS did not correlate with GA, preterm status, cardiac lesion subtype, birth factors, intraoperative factors, or different vendor MRI/field strength. There was no correlation between acquired brain injury patterns and BDS. BDS did correlate with developmental delay and poor expressive language development. There was no correlation between preoperative or postoperative status and BDS score. The BDS also correlated with age at surgery, increased length of hospitalization, and need for G-tube placement. A more detailed evaluation of factors surrounding feeding and individual brain dysplasia parameters found correlations between lack oral feeding prior to neonatal hospital discharge and dysplasia of multiple brain areas as well as the BDS, and additionally with hospital length of stay, which is likely the sequala of feeding dysfunction. Interestingly, swallowing dysfunction was associated with dysplasia of the brain stem. Of note, there are other possible confounding factors like anesthetic exposure and other clinical factors related to the hospital stay that we did not examine in our study, that could potentially impact brain neurogenesis and hence the BDS score.

An important component of our paralimbic-related subcortical BDS scoring system is the hippocampus. We found a consistent pattern of dysmaturation in the hippocampus, including severe forms of hippocampal aplasia or dysplasia in both mice and human subjects. Research has indicated that reductions in hippocampal volume has functional correlates in adolescents and adults, resulting in an increased rate of neurodevelopmental impairment [65,66]. Fontes et al. found that hippocampal shape and volumetric abnormalities found within CHD subjects can be predictive of impaired executive function. It is possible that the hippocampus is particularly vulnerable to early injury in CHD individuals as it is a brain region particularly susceptible to injury related to hypoxemia or hemodynamic instability. Latal et al. found that reductions in hippocampal volume correlated with reductions in total IQ, working memory, and verbal comprehension. Lastly, one study looking at maternal stress found that reductions in hippocampal volumes are present in utero, consistent with previous volumetric findings of CHD [67]. Wu et al. concluded that universal screening looking at maternal stress is important, as early identification of hippocampal abnormalities is imperative.

Another important component of our paralimbic-related subcortical scoring system was the cerebellum. Cerebellar dysfunction has been linked with adverse neurodevelopmental outcomes [68,69,70]. Stoodley found that cerebellar damage or malformation present in early stages of development to be more detrimental than when obtained in adulthood and theorized that injuries could affect cerebral–cerebellar circuits that are crucial to development and learning [69]. Zwicker et al. found that preterm infants with exposure to morphine in the neonatal period had decreased cerebellar volumes and worse neurodevelopmental outcomes [70]. Additionally, our group has previously shown that CHD children with reduced cerebellar volumes scored worse in tests for working memory, inhibitory control, and mental flexibility [71]. We also demonstrated that the superior surface of the cerebellum, primarily composed of the posterior lobe and the midline vermis, is an area particularly susceptible to alterations in morphology, indicating possible regions specifically affected by dysmaturation in CHD [23]. Previous literature in combination with our BDS findings show the importance of the cerebellum toward neurodevelopment and suggest that insults or injury affecting proper cerebellar development may impact neurodevelopmental outcomes. Figure 4 demonstrates some of the commonalities seen between the mouse and human cerebellum; of note are the abnormal number of cerebellar fissures and small size.

An important subcortical structure that was noted to be abnormal in the human BDS score volumetric correlation analysis and in the preclinical modeling was the striatum. In infancy, striatal connectivity is related to later motor and affective outcomes [72,73,74], with previous research suggesting there are abnormalities in CHD [75], potentially linking disrupted development with future behavior. The striatum forms a neural network with indirect and direct connectivity with other subcortical paralimbic components of the BDS score including the cerebellum, hippocampus, and olfactory bulb. As such the developing striatum is part of a dense, interconnected network that supports motor function, reward processing, and cognition [76,77], with circ uitry specialized for affective-based learning and olfactory reward. Supported by work in animal models and adults, the olfactory bulb directly projects to the olfactory tubercle [78], a part of the ventral striatum, specialized for olfaction and containing significant dopamine neurons [79,80]. Together with innervations to the striatum from the hippocampus and amygdala [81,82,83,84], the ventral striatum has been suggested to represent odor valence cues and instigate goal-directed behavior [78], facilitated by additional input from piriform cortex and prefrontal cortex [85,86,87]. Output from the ventral striatum proceeds to the pallidum and then the thalamus, which then closes the feedback loop with prefrontal cortex [88,89]. An important structure in this loop is the orbitofrontal cortex, which plays an important role in maintaining changing odor representations but also more broadly in decision making and stimulus evaluation [90]. Integrated into these loops are other subcortical structures, including the cerebellum, which also has a significant role in movement and more recently emphasized role in cognition and language [91]. Through the thalamus and pons, the cerebellum indirectly projects to putamen, pallidum, and the subthalamic nucleus [92,93]. These outputs may be topographically organized, converging on both the ventral striatum and frontal areas, with suggested involvement in appetitive reward [94] and strategic motor learning [95]. Disruption of this interconnected network is likely captured by BDS scores, which are then sensitive to striatally relevant neurodevelopmental outcomes.

Higher BDS in the CHD cohort was correlated with known genetic abnormalities in the human infants, suggesting a genetic mechanism might also drive these subcortical dysplastic structural findings. Concordant with this, we also found a similar spectrum of paralimbic and subcortical abnormalities exists between human and *Ohia* mutants, suggesting a common genetic mechanistic etiology. Within the *Ohia* mouse model, the two main driver genes were *Sap130* and *Pcdha9*, and while both genes were necessary to have cardiac phenotype, both genes were not required to have neuroanatomic abnormalities. Additionally, *Sap130* mutations presented with a more severe brain phenotype compared to *Pcdha9* mutations. This is interesting as knock-out mice for *Sap130* are embryonically lethal whereas *Pcdha9* knock-out mice are viable, suggesting that *Sap130* may have a more upstream effect whereas *Pcdha9* is more downstream [96]. *Sap130* functions as a subunit of the histone deacetylase-dependent co-repressor complex, which has roles in transcriptional regulation and has been specifically mentioned to be required for recovery from hypoxia [97]. *Pcdha9* is a member of the protocadherin alpha gene cluster, which are important plasma membrane proteins for cell–cell connections, neural projects, and synapse formation [98]. Interestingly, both genes show overlap in connectomes with chromatin and histone modification pathways [43].

Alterations in histone-modifying pathways have been linked to various forms of CHD [99,100,101]. It is likely that there are many other genes and gene pairings that could cause a similar neurodevelopmental phenotype as it is possible the main driver could be epigenetic dysregulation resulting in altered differential expression of genes required for signaling and migration. Incorrect timing for migrating neural cells might explain why preterm infants and CHD infants look phenotypically similar in regard to neurodevelopment and impact the BDS (particularly the olfactory bulb and hippocampus, which undergo early developmental neurogenesis). Looking at the genetics of the preclinical mouse model could not be conclusive as to the etiology of the human CHD neurodevelopmental issues; however, we demonstrate spectra of cardiac and brain anatomical abnormalities associated with the different genetic combinations of the *Ohia* mouse, parallel to those seen in human subjects.

CHD subjects have a documented risk of poor neurodevelopmental outcomes although the specific mechanism is unknown [102,103,104,105,106]. Additionally, neurodevelopmental impairments can continue to affect individuals well past infancy and into adolescence and early adulthood [107,108,109,110,111,112]. There are two main hypotheses with regard to the origin of injury to the brain and subsequent neurodevelopmental deficits that arise from the injury: intrinsic patient factors including genetics or altered substrate delivery due to the cardiac malformation. There have been studies looking at the heritability of CHD to determine the genetic causes, which resulted in the finding that CHD most likely has a genetic component. CHD is particularly common in genetic syndromes caused by aneuploidy defects, ranging anywhere from 20–100% of individuals with trisomies 13, 18, and 21 as well as and monosomy X [113], though these syndromic cases account for a relatively low amount of the total CHD population. Additionally, rates of CHD are increased when there are abnormal chromosomal structural syndromes such as deletions, insertions, and copy number variants [114]. Known de novo single-nucleotide polymorphisms account for approximately 10% of CHD cases, roughly the same amount that can be attributed to environmental factors [114,115]. However, the vast majority of CHD individuals do not have known origin of their cardiac defect nor their neurodevelopmental abnormalities. To overcome this missing data, work has begun looking at previously uncharacterized de novo mutations in mice, singling out genes with human homologs and which have high expression in the heart. When looking at only the homologous genes, researchers found that between CHD and control individuals, there was no significant difference between rates of generic de novo mutations; however, when comparing genes with high cardiac expression, CHD subjects had much higher rates of mutations in protein-altering mutations [116]. This recent work into the genetic origins of CHD has shown that even when the cause of the insult is unknown, the cause of CHD is likely genetic, possibly through the alteration of genes that affect similar downstream pathways.

Whether the genetic insult is the main driver or whether the mutations leave the individual susceptible to other forms of injury is unknown. Our group has previously looked at how the developing subcortical paralimbic-related structures of the brain can be linked to energy metabolism and found that subcortical morphological differences were not likely linked to prematurity or white matter injury alone, but that there was a linkage between brain metabolism and subcortical structural morphometry [55]. Stemming from these findings, we believe that subplate vulnerability in the prenatal and immediate post-natal period can be informative to understanding the subcortical dysmaturation seen in CHD infants. Within CHD individuals, it has been shown that preoperatively they have volumetric abnormalities present in the cerebellum and brainstem [17] and additionally that abnormal cerebellar growth has been linked with worse outcomes in expressive communication and the adaptive development quotient in CHD individuals [13]. Furthermore, there is an established link between subcortical morphological abnormalities and white matter vulnerability, which can lead to injury and eventual worse neurodevelopmental outcomes [18,21].

With the mounting evidence of the importance of subcortical structures for proper brain development, and the subcortical structures’ relationship to proper cortical development, inclusion of subcortical structures into our scoring system is necessary to characterize the entire picture of brain dysplasia. With nearly half of CHD cases still without a known genetic origin, identifying subtle cases of neurodevelopmental abnormalities in CHD individuals can better help us understand the downstream effects of CHD on brain development, ultimately furthering knowledge of the interplay between CHD and neurodevelopmental outcomes. Importantly, early interventional strategies have shown promise in helping CHD individuals with potential neurocognitive difficulties [117] and early identification of the most vulnerable through proper screening is necessary to identify and target therapies toward highest risk individuals. Future work is needed to develop the BDS utilizing large neuroimaging registries of clinical and research CHD subjects. Future scalability research is also needed to determine if the BDS could be integrated into existing clinical workflows and what resources or training clinicians would need to adopt this new scoring system.

### Limitations

Our study does have some important limitations. The preterm and term CHD cohorts that we studied were heterogenous related to heart lesion subtypes. Also, neurodevelopmental outcomes were only available for a small subset of our primary cohort. During this study, only a limited number of socio-demographic factors were prospectively collected, and therefore analysis using these factors was unable to be carried out and future work correlating BDS score with pediatric/adolescent neurocognitive outcomes in relation to socio-demographic factors is needed. Our multi-site approach with different scanner platforms and vendors can lead to interscanner variance, but our secondary analyses did not find a correlation between scanner vendor or field strength and brain dysplasia score. Future work employing retrospective machine learning techniques like the empirical Bayes technique (Combat) can be used to reduce interscanner variance. Importantly, we were not able to measure extra-axial CSF fluid in the ECM imaging of the mouse mutants, which is an important component of the BDS score. Complete removal of CSF and infiltration of paraffin could have altered CSF volumes or the ventricle shape. With this in mind, we did not feel comfortable labeling these brain regions as containing CSF and instead labeled them as extra-axial space. As such, some of the preclinical brain regions were omitted from the analysis, resulting in missing data points.

## Figures and Tables

**Table 1:** Incidence of Subcortical Brain Dysmaturation in Preterm/Term CHD and Controls.

BDS Abnormalities (Dichotomous)	Preterm CHD (*n* = 69)	Preterm Non-CHD (*n* = 51)	Comparison	Term CHD (*n* = 263)	Term Control (*n* = 93)	Comparison
	Number of Subjects with Abnormality (*%*)		*p*-Value	Number of Subjects with Abnormality (*%*)		*p*-Value
Bilateral Cerebellar Hemispheric Hypoplasia	13 (18.84%)	1 (1.96%)	**0.0049**	30 (11.41%)	0 (0.00%)	**0.0006**
Bilateral Cerebellar Hemispheric Dysplasia	7 (10.14%)	1 (1.96%)	0.0607	15 (5.70%)	0 (0.00%)	**0.0133**
Cerebellar Vermis Hypoplasia	18 (26.09%)	3 (5.88%)	**0.0040**	58 (22.05%)	0 (0.00%)	**<0.0001**
Cerebellar Vermis Dysplasia	9 (13.04%)	0 (0.00%)	**0.0049**	23 (8.75%)	0 (0.00%)	**0.0019**
Dichotomized Cerebellar Composite	26 (37.68%)	3 (5.88%)	**<0.0001**	75 (28.52%)	0 (0.00%)	**<0.0001**
Right Olfactory Bulb	24 (34.78%)	4 (7.84%)	**<0.0001**	127 (48.29%)	8 (8.60%)	**<0.0001**
Left Olfactory Bulb	25 (36.23%)	4 (7.84%)	**<0.0001**	126 (47.91%)	9 (9.68%)	**<0.0001**
Right Olfactory Sulcus	23 (33.33%)	1 (1.96%)	**<0.0001**	109 (41.44%)	3 (3.23%)	**<0.0001**
Left Olfactory Sulcus	23 (33.33%)	1 (1.96%)	**<0.0001**	109 (41.44%)	2 (2.15%)	**<0.0001**
Dichotomized Olfactory Composite	28 (40.58%)	4 (7.84%)	**<0.0001**	127 (48.29%)	9 (9.68%)	**<0.0001**
Hippocampus	34 (49.28%)	11 (21.57%)	**0.0019**	117 (44.49%)	3 (3.23%)	**<0.0001**
Corpus Callosum	10 (14.49%)	0 (0.00%)	**0.0045**	26 (9.89%)	0 (0.00%)	**0.0016**
Choroid Plexus	30 (43.48%)	3 (5.88%)	**<0.0001**	108 (41.06%)	8 (8.60%)	**<0.0001**
Brainstem	10 (14.49%)	0 (0.00%)	**0.0045**	25 (9.51%)	0 (0.00%)	**0.0020**
**BDS Abnormalities (Categorical)**	**Mean (Standard Error)**		***p*-Value**	**Mean (Standard Error)**		***p*-Value**
Supratentorial Extra-Axial Fluid	0.9839 (0.0989)	0.6000 (0.1069)	0.0075	**0.8071 (0.0495)**	0.3516 (0.0527)	**<0.0001**
Cerebellar Composite	0.6812 (0.1121)	0.0980 (0.0578)	<0.0001	**0.4791 (0.0536)**	0.0000 (0.0000)	**<0.0001**
Olfactory Composite	1.6912 (0.2693)	0.1961 (0.1011)	<0.0001	**2.0229 (0.1452)**	0.2418 (0.0817)	**<0.0001**
Subcortical BDS Composite	4.3188 (0.4043)	1.1569 (0.2046)	<0.0001	**4.1141 (0.2210)**	0.6989 (0.1268)	**<0.0001**

Note: Bolded values indicate statistically significant results.

**Table 2:** Correlation of Brain Dysplasia Score (BDS) with Demographic Factors (entire cohort: Univariate Analysis).

Dependent	Independent	N-Used	R-Square	*p*-Value	Estimate
BDS	Sex	490	0.000026	0.9096	−0.0359
BDS	GA	487	0.003619	0.1851	0.058461
BDS	PCA	489	0.001664	0.368	0.011933

**Table 3:** Correlation of Brain Dysplasia Score (BDS) with Demographic Factors (CHD cohort, Univariate Analysis).

Dependent	Independent	N-Used	R-Square	*p*-Value	Estimate
BDS	Sex	344	0.008447	0.0887	0.680037
BDS	Genetic Abnormality	252	0.045926	**0.0006**	−1.66277
BDS	GA	341	0.00002	0.9352	−0.00562
BDS	PCA	343	0.002649	0.3419	0.017322
BDS	Birth Weight	248	0.000337	0.7737	−9.1 × 10^−5^
BDS	Pre/Post operative status	343	0.000662	0.6349	−0.18132
BDS	Term/Preterm	343	0.000651	0.6378	−0.23214

Note: Bolded values indicate statistically significant results.

**Table 4:** Correlation of Brain Dysplasia Score (BDS) with Demographic Factors in (CHD cohort, Multivariate Analysis).

			Independent		Term/Preterm		Pre/Post	
Dependent	N-Used	R-Square	*p*-Value	Estimate	*p*-Value	Estimate	*p*-Value	Estimate
Sex	343	0.009957	0.0854	0.6942	0.7409	−0.1633	0.5872	−0.2074
Genetic Abnormality	252	0.048769	**0.0006**	−1.6806	0.3971	−0.4741	0.8911	−0.0617
GA	341	0.002196	0.6323	0.059	0.5401	−0.5386	0.5487	−0.2303
PCA	343	0.003471	0.3867	0.0168	0.6152	−0.2489	0.8939	−0.0543
Birth Weight	248	0.027317	0.4891	0.0002	0.0368	−1.4958	0.0878	−0.7169

Note: Bolded values indicate statistically significant results.

**Table 5:** Correlation of Brain Dysplasia Score, Brain Injury, and Cortical Maturation Score (TMS).

				Main Independent Factor		PCA	
	N-Used	Model p	R-Square	*p*-Value	Estimate	*p*-Value	Estimate
Hemorrhage	340	0.9876	0.000001	0.9876	0.010146	—	—
Infarction	341	0.604	0.000794	0.604	0.345161	—	—
Hypoxic ischemic injury	328	0.6111	0.000794	0.6111	−0.55119	—	—
Punctate white matter lesion	342	0.8922	0.000054	0.8922	0.071424	—	—
Any brain injury, dichotomized	344	0.5548	0.001021	0.5548	0.243539	—	—
Brain injury composite score	344	0.9042	0.000042	0.9042	−0.03758	—	—
Maturation, Cortical Fold	332	**0.0016**	0.029645	0.0016	−0.7302	—	—
Maturation, Frontal Cortex	332	**<0.0001**	0.061511	<0.0001	−0.80238	—	—
Maturation, Insular Cortex	332	**<0.0001**	0.067464	<0.0001	−1.01932	—	—
Maturation, Cortical Fold & PCA	331	**0.0044**	0.032554	0.001	−0.78513	0.3255	0.02414
Maturation, Frontal Cortex & PCA	331	**<0.0001**	0.067676	<0.0001	−0.86858	0.1531	0.034607
Maturation, Insular Cortex & PCA	331	**<0.0001**	0.07442	<0.0001	−1.10484	0.146	0.035021

Note: Bolded values indicate statistically significant results.

**Table 6:** Association Between Brain Dysplasia Score and Term Infant Regional Brain Volumes by CHD Status.

Normalized Volumes (*n* = 105)	Correlation with BDS (Term CHD and Term Control Cohort Combined)	Comparison between Term CHD and Term Control Cohorts
	*p*-Value (Correlation)	*p*-Value (Direction)
Right Hippocampus	0.0749 (N.S.)	0.1476 (N.S.)
Left Hippocampus	0.1329 (N.S.)	0.3784 (N.S.)
Right Cerebellum	0.0535 (N.S.)	0.1476 (N.S.)
Left Cerebellum	**0.0433** (**−**)	0.1887 (N.S.)
Cerebellum	**0.0299** (**−**)	**0.0087** (**CHD > CON**)
Cerebrospinal Fluid (CSF)	0.0517 (N.S.)	**0.0026** (**CHD > CON**)
Infratentorial CSF	0.2259 (N.S.)	0.9300 (N.S.)
Intraventricular CSF	**<0.0001** (**+**)	0.7398 (N.S.)
Supratentorial CSF	**0.0008** (**+**)	0.3535 (N.S.)
Deep Grey Matter	**0.0138** (**−**)	0.4877 (N.S.)
White Matter	0.4550 (N.S.)	0.6066 (N.S.)
Cerebral Cortex	0.1687 (N.S.)	**0.0016** (**CHD > CON**)
Brainstem	**0.0391** (**−**)	0.1311 (N.S.)

Note: Bolded values indicate statistically significant results.

**Table 7:** Correlation between Brain Dysplasia Score and Clinical Feeding Outcomes.

BDS Abnormalities (Clinical Outcome Correlates)	Lack of PO Feeds Prior to Discharge *p*-Value	Length of Hospitalization *p*-Value
Hippocampus	**0.0025**	**0.0090**
Right/Left Olfactory Bulbs	**0.0090/0.0050**	**0.0400/0.0300**
Right/Left Olfactory Sulci	**0.0070/0.0070**	**0.0700/0.0700**
Extra-Axial Fluid	**0.0030**	**<0.0001**
Bilateral Cerebellar Hemispheres	**0.0037**	**<0.0001**
Cerebellar Vermis	**0.0001**	**0.0002**
Brainstem	**<0.0001**	N.S.
Corpus Callosum	**0.0010**	**0.0100**
Choroid Plexus	**0.0100**	**0.0020**
*Total Composite*	**0.0001**	**0.0020**

Note: Bolded values indicate statistically significant results.

**Table 8:** Correlation between Brain Dysplasia Score and 15–18-Month Neurodevelopmental Outcomes.

Neurodevelopmental Outcomes (*n* = 90) (Total BDS Correlates)	Non-Parametric			Parametric
	Wilcoxon Mann–Whitney *p*-Value (Normal Approx.)	Wilcoxon Mann–Whitney *p*-Value (T Approx.)	Kruskal–Wallis *p*-Value (χ2)	*t*-Test *p*-Value
Expressive Language Below Mean	**0.0160**	**0.0190**	**0.0160**	**0.0047**
Language Composite Below Mean	0.2810	0.2840	0.2790	0.2837
Language Delay	**0.0380**	**0.0420**	**0.0370**	**0.0181**
Developmental Delay (delayed in ≥ 1 domains)	**0.0280**	**0.0303**	**0.0272**	**0.0284**
Global Developmental Delay (delayed in ≥ 2 domains)	0.1070	0.1110	0.1060	0.1005

Note: Bolded values indicate statistically significant results.

**Table 9:** Incidence of Brain Dysplasia Score and Subcomponents between *Ohia* and Wild type Mouse Mutants.

Structure	*Ohia* Average	Wild Type Average	*p* Value ^†^
BDS Total	**4.42**	0	**<0.001**
Cerebellar Fissures	**1.5**	4.4	**<0.001**
	***Ohia* Incidence**	**Wild Type Incidence**	*p* value *
BDS Dichotomized	**55/69, (79.71%)**	0/10, (0.00%)	**<0.001**
BDS Hippocampus or Cerebellum	**58/69, (84.06%)**	0/10, (0.00%)	**<0.001**
Aplastic Hippocampus	8/69, (11.59%)	0/10, (0.00%)	0.384
Hypoplastic Hippocampus	14/68, (20.59%)	0/10, (0.00%)	0.223
Dysplastic Hippocampus	**44/68, (64.71%)**	0/10, (0.00%)	**0.002**
Combination Hippocampus	22/69, (31.88%)	0/10, (0.00%)	0.133
Hypoplastic Cerebrum	13/68, (19.12%)	0/10, (0.00%)	0.244
Dysplastic Cerebrum	**43/68, (63.24%)**	0/10, (0.00%)	**0.003**
Aplastic Cerebellum	2/69, (02.90%)	0/10, (0.00%)	0.678
Hypoplastic Cerebellum	9/67, (13.43%)	0/10, (0.00%)	0.345
Dysplastic Cerebellum	**49/67, (73.13%)**	0/10, (0.00%)	**<0.001**
Combination Cerebellum	11/69, (15.94%)	0/10, (0.00%)	0.325
Aplastic Left Olfactory Bulb	**33/69, (47.83%)**	0/10, (0.00%)	**0.024**
Aplastic Right Olfactory Bulb	**34/69, (49.28%)**	0/10, (0.00%)	**0.02**
Hypoplastic Left Olfactory Bulb	9/67, (13.43%)	0/10, (0.00%)	0.345
Hypoplastic Right Olfactory Bulb	7/65, (10.77%)	0/10, (0.00%)	0.404
Dysplastic Left Olfactory Bulb	2/67, (2.99%)	0/10, (0.00%)	0.673
Dysplastic Right Olfactory Bulb	0/65, (00.00%)	0/10, (0.00%)	—
Combination Left Olfactory Bulb	**42/69, (60.87%)**	0/10, (0.00%)	**0.006**
Combination Right Olfactory Bulb	**41/69, (59.42%)**	0/10, (0.00%)	**0.007**
Hypoplastic Brainstem	0/66, (00.00%)	0/10, (0.00%)	—
Dysplastic Brainstem	20/67, (29.85%)	0/10, (0.00%)	0.12
Hypoplastic Midbrain	0/67, (00.00%)	0/10, (0.00%)	—
Dysplastic Midbrain	18/67, (26.87%)	0/10, (0.00%)	0.148

Note: Bolded values indicate statistically significant results. ^†^ Student’s *t*-test was used to compare Cerebellar Fissure count and BDS Total Score. * Chi-square analysis was used to compare individual structures as well as BDS Dichotomized and BDS Hippocampus or Cerebellum.

**Table 10:** Relationship between Brain Dysplasia Scores/Cerebellar Fissures and Cardiac Lesion Subtypes in *Ohia* CHD/HLHS Preclinical Groups.

	CHD vs. No CHD			Single vs. Biventricular Morphology			Conotruncal vs. Non Conotruncal			Cyanotic vs. Acyanotic			Arch Obstruction vs. No Obstruction		
	CHD	No CHD	*p* Value *	Single	Double	*p* Value *	Conotruncal	Non Conotruncal	*p* Value *	Cyanotic	Acyanotic	*p* Value *	Obstruction	No Obstruction	*p* Value *
Cerebellar Fissures	1.17 ± 1.55	3.14 ± 1.67	**<0.001**	1.50 ± 2.12	1.16 ± 1.55	0.86	1.13 ± 1.92	1.22 ± 1.36	0.88	1.16 ± 1.80	1.19 ± 1.39	0.96	0.81 ± 0.98	1.48 ± 1.87	0.13
BDS Total	5.43 ± 2.22	2.73 ± 2.46	**<0.001**	7.00 ± 2.00	5.32 ± 2.20	0.21	5.27 ± 2.12	5.33 ± 2.46	0.93	5.63 ± 2.34	5.29 ± 2.17	0.61	5.87 ± 1.69	5.00 ± 2.60	0.18

Note: Bolded values indicate statistically significant results. * Student *t*-test used to calculate difference between population values within cerebellar fissures and Total Brain Dysmaturation Score (BDS).

**Table 11:** Relationship of Genotype to Incidence of Subcortical Abnormalities used to Derive BDS score in CHD/HLHS Preclinical Groups.

Group	A ^†^	B ^‡^	C ^§^	D ^||^	E ^¶^	F ^#^
N Total	24	8	7	4	0	20
Olfactory bulb abnormality	18 (75%)	6 (75%)	6 (85.7%)	4 (100%)	0	9 (45%)
Cerebellar abnormality	21 (87.5%)	7 (87.5%)	7 (100%)	4 (100%)	0	13 (65%)
Hippocampus abnormality	21 (87.5%)	7 (87.5%)	6 (85.7%)	4 (100%)	0	13 (65%)
Brainstem abnormality	10 (42%)	2 (25%)	0 (0%)	2 (50%)	0	6 (30%)

^†^ Group A: N 24, Genotype: Pcdha9(m/m) Sap130(m/m). ^‡^ Group B: N 8, Genotype: Pcdha9(m/+) Sap130(m/m). ^§^ Group C: N 7, Genotype: Pcdha9(+/+) Sap130(m/m). ^||^ Group D: N 4, Genotype: Pcdha9(m/m) Sap130(m/+). ^¶^ Group E: N 0, Genotype: Pcdha9(m/m) Sap130(+/+). ^#^ Group F: N 27, Genotype: Pcdha9(m/+) or (+/+) Sap130(m/+) or (+/+).

**Table 12:** Relationship of Genotype to BDS Score in CHD/HLHS Preclinical Groups.

	ABC ** vs. WT ^‡‡^			ABC ** vs. F ^#^			AB ^††^ vs. F ^#^			A^†^ vs. C ^§^			B ^‡^ vs. D ^||^		
Structure	ABC Median	WT Median	*p* Value *	ABC Median	F Median	*p* Value *	AB Median	F Median	*p* Value *	A Median	C Median	*p* Value *	B Median	D Median	*p* Value *
BDS Hippocampus or Cerebellum	1	0	**<0.001**	1	1	**0.008**	1	1	**0.021**	1	1	0.583	1	1	0.46
BDS Total	6	0	**<0.001**	6	5.5	0.227	6	5.5	0.251	6	5	0.56	6	6.5	0.558

Note: Bolded values indicate statistically significant results. * Non-parametric Mann–Whitney U test used to analyze differences in continuous Brain Dysmaturation Score. ^†^ Group A: N 24, Genotype: Pcdha9(m/m) Sap130(m/m). ^‡^ Group B: N 8, Genotype: Pcdha9(m/+) Sap130(m/m). ^§^ Group C: N 7, Genotype: Pcdha9(+/+) Sap130(m/m). ^||^ Group D: N 4, Genotype: Pcdha9(m/m) Sap130(m/+). ^#^ Group F: N 27, Genotype: Pcdha9(m/+) or (+/+) Sap130(m/+) or (+/+). ** Group ABC: N 39, Genotype: Sap130(m/m), Pcdha9 (*/*). ^††^ Group AB: N 32, Genotype: Sap130(m/m), Pcdha9 (m/*). ^‡‡^ Group WT: N 10.

## Data Availability

Preclinical mouse data can be made available upon request to corresponding author. Clinical data cannot be made public due to privacy issues, but limited data can be made available upon special request to the corresponding author with justification for the data request.

## Supplementary Materials

The following supporting information can be downloaded at https://www.mdpi.com/article/10.3390/jcm13195772/s1: Table S1: Incidence of Brain Injury in Human Infant Term and Preterm CHD, Table S2: Comparison of Cortical Maturation Score (TMS) between CHD and Controls Infants (Preterm and Term), Table S3: Inter-rater Reliability (Kappa) for Brain Dysplasia Score Between Two Pediatric Neuroradiologists, Table S4A: Association Between Innate Factors, Heart Lesion Subtypes and Brain Dysplasia Score in Term CHD, Table S4B: Association Between Preoperative Risk Factors and Brain Dysplasia Score, Table S4C: Human Infant Association Between Intraoperative Factors and Brain Dysplasia Score, Table S4D: Human Association Between Post-Operative Clinical Risk Factors and Brain Dysplasia Score, Table S5: Association Between Field Strength and Brain Dysplasia Score, Table S6: Brain Dysplasia Score within *Ohia* Mouse Mutant Cohort: Comparison between Cardiac Lesion Subgroups, Table S7: *Ohia* Mouse Mutant Cohort Regional Cerebral Volumes Compared to Wild Type (WT), Table S8: Non-normalized Regional Cerebral Volumes of *Ohia* CHD cohorts: Comparison of Cardiac Lesion Subgroups, Table S9: Normalized Regional Cerebral Volumes of *Ohia* CHD cohorts: Comparison of Cardiac Lesion Subgroups, Table S10: Overview of *Ohia* Mutant Model Genotype Groups Studies: Congenital heart disease lesions and Holoprosencephaly Incidence, Table S11: Genotype/Brain Dysplasia Score, Table S12: Genotype/Regional Brain Volumes, Table S13: Genotype/Regional Brain Volume, Figure S1: Components of Human Brain Dysplasia Scoring Criteria, Figure S2: Components of Composite Brain Injury Score, Figure S3: Flow Diagram for NeBSS, a Semi-automated Neonatal Segmentation Pipeline, Figure S4: Flow Diagram of Sample Preparation, ECM Analysis, and Volumetric Analysis, Figure S5: Necropsy Among Mutant Mice Showed Variety and Differing Degree of Severity of Malformations, Figure S6: Individual Structures Showed a Range of Abnormality in Screen Mice, Figure S7: Varying Degree of Cerebral Abnormalities Present in Screen Mice, Figure S8: Crispr/Cas Figure Mutant Animals Showed Similar Phenotype to Screen Mice, Figure S9: Holoprosencephaly and Hippocampal Malformations Seen in Human and Mouse.

## Abbreviations and Acronyms

BDS: brain dysplasia score, CHD: congenital heart disease, CSF: cerebrospinal fluid, ECM: episcopic confocal microscopy, GA: gestational age, HLHS: hypoplastic left heart syndrome, MRI: magnetic resonance imaging, PCA: post-conceptional age, TMS: total maturation score.
