# Supplementary material for: Validation of a Paralimbic-Related Subcortical Brain Dysmaturation MRI Score in Infants with Congenital Heart Disease
Source: J Clin Med. 2024 Sep 27;13(19):5772. doi: 10.3390/jcm13195772 (PMC11476423; doi:10.3390/jcm13195772)
Supplement: Supplementary file 1 [file jcm-13-05772-s001.zip › jcm-3042431-supplementary.pdf]

Supplemental Table S1: Incidence of Brain Injury in Human Infant Term and Preterm CHD

Injury (Dichotomous)	**Preterm CHD (n=69)**	**Preterm Non-CHD (n=51)**	**Comparison**	**Term CHD (n =265)**	**Term Controls (n=93)**	**Comparison**
	**Number of Subjects with Injury (%)**		**p-value**	**Number of Subjects with Injury (%)**		**p-value**
Hemorrhage	10 (14.71%)	9 (17.65%)	0.6647	19 (7.20%)	1 (1.09%)	**0.0284**
Focal Infarct	6 (8.82%)	0 (0.00%)	**0.0295**	23 (8.68%)	0 (0.00%)	**0.0035**
Hypoxic Ischemic Injury	2 (3.03%)	0 (0.00%)	0.2144	8 (3.15%)	0 (0.00%)	0.1038
Punctate White Matter Lesions	12 (17.39%)	2 (3.92%)	**0.0231**	40 (15.09%)	0 (0.00%)	**<0.0001**
Dichotomized Injury Composite	25 (36.23%)	11 (21.57%)	0.0831	74 (27.92%)	1 (1.08%)	**<0.0001**
Injury (Categorical)	**Mean *(Standard Error)***		**p-value**	**Mean *(Standard Error)***		**p-value**
Injury Composite	0.434783 *(0.075797)*	0.215686 *(0.058166)*	0.0581	0.339623 *(0.037319)*	0.010753 *(0.010753)*	**<0.0001**

Supplemental Table S2: Comparison of Cortical Maturation Score (TMS) between CHD and Controls Infants (Preterm and Term)

BDS Maturation (Categorical)	Preterm CHD (*n* = 69)	Preterm Non-CHD (*n* = 51)	Comparison	Term CHD (*n* = 260)	Term Control (*n* = 93)	Comparison
	**Mean *(Standard Error)***		**p-value**	**Mean *(Standard Error)***		**p-value**
Occipital Cortex: Cortical Folding	3.0435 *(0.1041)*	2.8627 *(0.1284)*	0.3095	2.9577 *(0.0515)*	2.7849 *(0.1473)*	0.6235
Frontal Cortex: Cortical Folding	2.5942 *(0.1359)*	2.4902 *(0.1735)*	0.6604	1.2366 *(0.0786)*	2.6731 *(0.0710)*	**<0.0001**
Insular Cortex: Cortical Folding	2.9855 *(0.1195)*	3.0980 *(0.1407)*	0.5044	3.1538 *(0.0537)*	4.3441 *(0.0692)*	**<0.0001**
Frontal Cortex: Dark Bands on T2	1.8986 *(0.1477)*	1.9020 *(0.1820)*	0.8021	2.2335 *(0.0738)*	3.6882 *(0.0865)*	**<0.0001**
Parietal Cortex: Dark Bands on T2	2.2899 *(0.1587)*	2.6863 *(0.2029)*	0.1345	2.8308 *(0.0746)*	3.9140 *(0.0423)*	**<0.0001**
Myelination: Dark Bands on T2	3.5735 *(0.1297)*	4.1176 *(0.1143)*	**0.0049**	4.0888 *(0.0603)*	5.4409 *(0.0725)*	**<0.0001**

Supplemental Table S3: Inter-rater Reliability (Kappa) for Brain Dysplasia Score Between Two Pediatric Neuroradiologists

	**κ (kappa) Reviewer 1**	**κ (kappa) Reviewer 2**
**Microcephaly Yes/No**	1	0.4167
**Macrocephaly Yes/No**	1	1
**Cerebellar R/L Hemispheric Hypoplasia**	1	0.6786
**Cerebellar R/L Hemispheric Dysplasia**	1	0
**Cerebellar Vermian Hypoplasia Yes/No**	0.708333	0.6434
**Cerebellar Vermian Dysplasia Yes/No**	1	0.2895
**Supratentorial Extra-axial fluid**	0.350962	0.0526
**Maturational Parameters: Cortical Folding**	0.18306	0
**Maturational Parameters: Frontal**	0.371257	0.3698
**Maturational Parameters: Insular Cortex**	0.310627	0.3413
**Dark bands on T2 (cellular): Frontal**	0.638365	0.3061
**Dark bands on T2 (cellular): parietal**	0.904959	0.2140
**Dark bands on T2 (cellular): Myelination [M]**	0.350427	0.0256

**Dark bands on T2 (cellular): Germinal Matrix**	0.480226	0
**Dysmorphometry: Right Olfactory Bulb**	0.893773	0.6346
**Left Olfactory Bulb**	0.843243	0.7373
**Right Olfactory Sulci**	1	0.6329
**Left Olfactory Sulci**	1	0.6949
**Micrognathia**	0.68272	0.4408
**Midface hypoplasia**	1	1
**Temporal bone abnormalities**	1	0.5385
**Orbital abnormality**	0.649351	0.4000
**Hippocampal abnormalities**	0.910217	0.5190
**Cortical thickness abnormalities**	1	0.4633
**Corpus callosum malformation**	0.888031	0.5957
**Corpus callosum volume abnormalities**	0.828402	0.3284
**Ventriculomegaly**	0.538462	0.3640
**Absent Septum pellucidum**	0.606335	0.9474
**Choroid plexus abnormality**	0.125628	0.4311
**Brainstem Dysplasia**	0.888031	0.5342
**Holoprosencephaly3**	1	1
**Interdigitation of medial gyri**	1	1
**Injury: Hemorrhage**	0.867925	0.7692
**Infarct**	1	1
**Hypoxic-ischemic central BG/thal/perirolandic injury pattern**	0.648649	1
**Puncate white matter lesion Yes/No**	0.626667	0.7805

Supplemental Table S4A: Association Between Innate Factors, Heart Lesion Subtypes and Brain Dysplasia Score in Term CHD

Clinical Characteristics<br>(Total Composite Correlates)	R^2	p-value<br>*(estimate)*
Birth Weight (g)	0.0007	0.8186 *(-0.0001)*
Birth Weight Percentile	0.0110	0.3538 *(-0.0107)*
Head circumference (cm)	0.0396	0.0785 *(0.1712)*
Head circumference Percentile	0.0302	0.1255 *(0.0175)*
Birth Length (cm)	0.0137	0.3140 *(0.0520)*
Birth Length Percentile	0.0001	0.9386 *(-0.0009)*

APGAR (1 minute)	0.0001	0.9417 *(-0.0142)*
APGAR (5 minutes)	0.0074	0.4670 *(0.1899)*
22q11 microdeletion	0.0294	0.1862 *(-1.7758)*
Single Ventricle	0.0052	0.5245 *(-0.4444)*
Aortic arch Obstruction	0.0055	0.5118 *(0.4616)*
Double Ventricle with Arch Obstruction	0.0091	0.4009 *(0.9202)*
Single Ventricle with Arch Obstruction	0.0018	0.7118 *(0.2764)*
d-Transposition of Great Arteries	0.0026	0.6547 *(0.3345)*
Conotruncal cardiac defect	0.0104	0.3683 *(0.6354)*
Altered fetal cerebral substrate delivery	0.0092	0.4069 *(0.9420)*
Altered fetal cerebral substrate delivery, severity score	0.0143	0.3114 *(1.2857)*
Heterotaxy	0.0219	0.1903 *(1.8667)*

Supplemental Table S4B: Association Between Preoperative Risk Factors and Brain Dysplasia Score

Preoperative Factors<br>(Total Composite Correlates)	*N with data*	*R^2*	p-value<br>*(estimate)*
Preoperative Arterial Blood Gas (ABG) pH	53	0.0316	0.2029 *(-8.3331)*
Preoperative ABG PaO2	51	0.0053	0.6130 *(-0.0106)*
Preoperative ABG Lactate (mmol/L)	63	0.0015	0.7598 *(0.1539)*
Preoperative Renal Dysfunction (CR > 1)	75	0.0000	0.9661 *(0.1351)*
Preoperative Hepatic Dysfunction (INR > 2)	65	0.0092	0.4485 *(-1.7460)*
Preoperative Inotrope Use	80	0.0008	0.8043 *(-0.1764)*
Age at Surgery (postnatal days)	78	0.0001	0.9393 *(-0.0009)*
Age at Surgery ≤7 days	78	**0.0503**	**0.0484 *(-1.4231)***
Age at Surgery (post conceptual age – weeks)	78	0.6598	0.4347 *(-3.0000)*

Supplemental Table S4C: Human Infant Association Between Intraoperative Factors and Brain Dysplasia Score

Intraoperative Factors (Total Composite Correlates)	*N with data*	*R^2*	p-value *(estimate)*
Bypass Procedure	90	0.0044	0.5348 *(0.6421)*
Bypass Time (minutes)	82	0.0070	0.4563 *(0.0046)*
Aortic Cross-Clamp Procedure	86	0.0087	0.3932 *(0.6134)*
Aortic Cross-Clamp Time (minutes)	65	0.0094	0.4430 *(0.0075)*
Circulatory Arrest / DHCA Procedure	86	0.0037	0.5802 *(0.3885)*
Circ arrest / DHCA Time (minutes)	73	0.0001	0.9205 *(0.0019)*

Supplemental Table S4D: Human Association Between Post-Operative Clinical Risk Factors and Brain Dysplasia Score

Clinical Risk Factors (Total Composite Correlates)	N with data	R^2	p-value *(estimate)*
ECMO 1st hospitalization	87	0.0365	0.0764 *(-1.6194)*
ECMO (days)	62	0.0251	0.2190 *(0.3209)*
Delayed Sternal Closure	90	0.0006	0.8263 *(0.1662)*
Number of Cardiac Surgeries Lifetime	90	0.0033	0.5908 *(-0.2136)*
Unplanned interventions 1st hospitalization	88	0.0023	0.6597 *(0.3063)*
Length of ICU stay (days)	90	0.0373	0.0681 *(0.0190)*
Length of Hospitalization (days)	90	**0.0447**	**0.0454 *(0.0180)***
Expired 1st hospitalization	90	0.0224	0.1596 *(-2.3023)*
CPR 1st hospitalization	90	0.0021	0.6678 *(0.6353)*
Seizures in ICU	90	0.0090	0.3748 *(-0.8289)*
Home anti-epileptics	90	0.0143	0.2610 *(-1.1600)*
Home with G-tube	90	**0.0611**	**0.0189 *(-1.9214)***
Home with tracheostomy / ventilator	90	0.0176	0.2131 *(-2.0407)*

Supplemental Table S5: Association Between Field Strength and Brain Dysplasia Score

**Brain Dysplasia Score: Abnormalities / Injury / Maturation**	**Satterthwaite**		**ANOVA**		**Satterthwaite**		**ANOVA**	
	**Preterm CHD & Control p-value**	**Term CHD & Control p-value**	**Preterm CHD & Control p-value**	**Term CHD & Control p-value**	**Preterm CHD p-value**	**Term CHD p-value**	**Preterm CHD p-value**	**Term CHD p-value**
Bilateral Cerebellar Hemispheric Hypoplasia	0.0992	0.6417	0.5888	**0.0126**	**0.0495**	0.6832	0.2660	0.1800
Bilateral Cerebellar Hemispheric Dysplasia	0.0901	**0.0004**	0.2167	**0.0002**	**0.0123**	**0.0005**	0.1048	**0.0001**
Cerebellar Vermis Hypoplasia	**0.0370**	0.0846	0.6773	**0.0019**	**0.0192**	0.6006	0.4229	0.1263
Cerebellar Vermis Dysplasia	**0.0033**	**0.0001**	**0.0245**	**0.0007**	**0.0033**	**0.0003**	0.0601	**0.0003**
Cerebellum Composite	0.8107	**0.0008**	0.4113	**0.0015**	0.5311	**0.0120**	0.7134	**0.0109**
Dichotomized Cerebellum Composite	0.6801	**0.0021**	0.3437	**0.0011**	0.3936	0.0664	0.6636	**0.0389**
Right Olfactory Bulb	0.9840	**0.0055**	0.7455	**<0.0001**	0.4382	0.3406	0.4817	**0.0435**
Left Olfactory Bulb	0.8473	**0.0055**	0.6288	**<0.0001**	0.5612	0.3012	0.5761	**0.0252**
Right Olfactory Sulcus	0.9673	**0.0007**	0.6053	**0.0023**	0.5073	0.1067	0.6688	0.1043
Left Olfactory Sulcus	0.9115	**0.0005**	0.5670	**0.0012**	0.5513	0.1067	0.7082	0.1043
Olfactory Composite	0.7447	**0.0011**	0.8922	**0.0001**	0.3158	0.0928	0.2464	**0.0221**
Dichotomized Olfactory Composite	0.8473	**0.0061**	0.6288	**<0.0001**	0.5612	0.3215	0.5761	**0.0348**
Hippocampus	0.5216	**0.0013**	0.6854	**<0.0001**	0.2794	0.2063	0.9440	**0.0125**
Corpus Callosum	**0.0398**	**0.0048**	**0.0172**	**0.0194**	0.0666	**0.0192**	**0.0428**	**0.0063**
Choroid Plexus	0.8474	**0.0070**	0.4794	0.6707	0.9468	0.1532	0.5233	0.6467
Brainstem	0.3111	**<0.0001**	0.3583	**<0.0001**	0.5286	**<0.0001**	0.6414	**<0.0001**
Supratentorial Extra-Axial Fluid	**0.0016**	**0.0157**	**0.0383**	**0.0001**	**0.0012**	0.3355	**0.0225**	**0.0327**
Hemorrhage	0.3347	0.8587	0.5106	0.7653	0.4820	0.3420	0.7880	0.5194
Infarct	0.5397	0.1225	1.0000	**0.0271**	0.7489	0.3461	0.6952	**0.0331**
Hypoxic Ischemic Injury	0.8730	0.1968	0.5849	0.1611	0.7639	0.3109	0.4337	0.0926
Punctate White Matter Lesions (PWM)	0.8257	**0.0266**	0.1972	0.3759	0.8279	0.1580	0.4210	0.4350
PWM: Laterality	0.5384	0.8623	0.7114	0.7785	0.5384	0.8623	0.7114	0.7785
PWM: Distribution	0.2889	0.3115	0.1885	0.8495	0.2889	0.3115	0.1885	0.8495
PWM: Lobes	0.9155	0.1419	0.1588	0.1552	0.9155	0.1419	0.1588	0.1552
PWM: Spatially Co-incident with Banding	.	**0.0261**	0.6887	**<0.0001**	.	0.3678	0.6887	**0.0330**
Injury Composite	0.7851	**0.0114**	0.8366	0.1274	0.5979	0.2012	0.9403	0.1086
Dichotomized Injury Composite	0.6479	**0.0088**	0.5743	0.2336	0.5135	0.2551	0.4066	0.0533
Occipital Cortex: Cortical Folding	**0.0341**	0.1383	0.5319	**0.0016**	0.0531	0.1043	0.8062	0.0986
Frontal Cortex: Cortical Folding	**0.0117**	**0.0256**	0.0558	**0.0003**	**0.0106**	0.6073	0.0611	0.5007
Insular Cortex: Cortical Folding	0.7842	**0.0055**	0.3428	**<0.0001**	0.4037	1.0000	0.7304	0.0507
Frontal Cortex: Dark Bands on T2	0.0828	**0.0466**	**0.0191**	**<0.0001**	0.1625	0.5799	**0.0477**	0.3573
Parietal Cortex: Dark Bands on T2	0.6331	0.8653	0.2648	**<0.0001**	0.7610	**0.0352**	0.3236	**0.0052**
Myelination: Dark Bands on T2	0.8579	**0.0001**	0.3030	**0.0357**	0.3318	0.4581	0.7365	0.2596
Germinal Matrix: Dark Bands on T2	0.1045	0.6032	0.2885	0.2352	**0.0326**	0.1555	0.0537	**0.0474**
Total Composite	0.5501	**<0.0001**	0.7548	**<0.0001**	0.1787	**0.0127**	0.5573	**0.0069**

** Welch–Satterthwaite t-test was used to test volume differences among individual structures.*

Supplemental Table S6A: Brain Dysplasia Score within Ohia Mouse Mutant Cohort: Comparison between Cardiac Lesion Subgroups

	**CHD vs. No CHD**				**Single vs. Biventricular Morphology**			
**Structure**	N	CHD	No CHD	P value *	N	Single	Double	P value *
**Aplastic Hippocampus**	68	8	0	0.050	49	0	8	0.534
**Hypoplastic Hippocampus**	68	10	4	0.868	48	1	9	0.310
**Combination Hippocampus**	69	43	9	0.059	49	2	41	0.955
**Dysplastic Hippocampus**	68	**33**	9	**0.043**	48	2	31	0.341
**Hypoplastic Cerebrum**	68	**13**	0	**0.008**	48	0	13	0.389
**Dysplastic Cerebrum**	68	**35**	6	**<0.001**	48	2	33	0.489
**Aplastic Cerebellum**	69	1	1	0.538	49	1	0	**<0.001**
**Hypoplastic Cerebellum**	67	6	2	0.707	47	1	5	0.112
**Combination Cerebellum**	69	42	9	0.818	49	2	**40**	**<0.001**
**Dysplastic Cerebellum**	67	**40**	7	**<0.001**	47	1	39	0.161
**Aplastic L. Olf. Bulb**	69	**30**	2	**<0.001**	49	2	28	0.260
**Hypoplastic L. Olf. Bulb**	67	7	2	0.553	47	0	7	0.556
**Combination L. Olf. Bulb**	69	**37**	4	**<0.001**	49	2	35	0.292
**Dysplastic L. Olf. Bulb**	67	2	0	0.345	47	0	2	0.767
**Aplastic R. Olf. Bulb**	69	**30**	2	**<0.001**	49	2	28	0.281
**Hypoplastic R. Olf. Bulb**	65	4	3	0.515	45	0	4	0.660
**Combination R. Olf. Bulb**	69	**34**	5	**<0.001**	49	2	32	0.301
**Dysplastic R. Olf. Bulb**	65	0	0	.	45	0	0	.
**Hypoplastic Brainstem**	66	0	0	.	46	0	0	.
**Dysplastic Brainstem**	67	**18**	2	**0.016**	47	2	16	0.069
**Hypoplastic Midbrain**	67	0	0	.	47	0	0	.
**Dysplastic Midbrain**	67	**17**	1	**0.006**	47	2	15	0.057
**BDS Dichotomized**	69	**45**	10	**<0.001**	49	2	43	0.635
**BDS Hippocampus or Cerebellum**	69	**45**	10	**<0.001**	49	2	43	0.675

** P value is calculated from two sample Student T-test*

Supplemental Table S6B: Brain Dysplasia Score within Ohia Mouse Mutant Cohort: Comparison between Cardiac Lesion Subgroups

	**Conotruncal vs. Non Conotruncal**				**Cyanotic vs. Acyanotic**				**Arch Obstruction vs. No Arch Obstruction**			
**Structure**	N	Cono.	Non Cono.	P value *	N	Cyanotic	Acyanotic	P value *	N	Obstr.	No Obstr.	P value *
**Aplastic Hippocampus**	49	3	5	0.723	49	3	5	0.839	49	6	2	0.144
**Hypoplastic Hippocampus**	48	2	8	0.425	48	4	6	0.977	48	4	6	0.488
**Combination Hippocampus**	49	14	29	0.900	49	18	25	0.819	49	22	21	0.313
**Dysplastic Hippocampus**	48	**10**	23	0.948	48	14	19	0.560	48	15	18	0.361
**Hypoplastic Cerebrum**	48	**1**	12	**0.037**	48	2	11	**0.037**	48	7	6	0.752
**Dysplastic Cerebrum**	48	**10**	25	0.668	48	13	22	0.828	48	18	17	0.365
**Aplastic Cerebellum**	49	0	1	0.498	49	1	0	0.232	49	1	0	0.332
**Hypoplastic Cerebellum**	47	2	4	0.909	47	3	3	0.618	47	2	4	0.424
**Combination Cerebellum**	49	13	29	0.692	49	17	**25**	0.240	49	23	**19**	0.957
**Dysplastic Cerebellum**	47	**12**	28	0.684	47	15	25	0.339	47	22	18	**0.048**
**Aplastic L. Olf. Bulb**	49	**12**	18	0.143	49	15	15	0.104	49	16	14	0.692
**Hypoplastic L. Olf. Bulb**	47	0	7	0.055	47	0	7	**0.018**	47	4	3	0.647
**Combination L. Olf. Bulb**	49	**12**	25	0.379	49	15	22	0.375	49	20	17	0.551
**Dysplastic L. Olf. Bulb**	47	0	2	0.341	47	0	2	0.243	47	1	1	0.976
**Aplastic R. Olf. Bulb**	49	**12**	18	0.202	49	15	15	0.164	49	16	14	0.493
**Hypoplastic R. Olf. Bulb**	45	0	4	0.152	45	0	4	0.076	45	2	2	0.964
**Combination R. Olf. Bulb**	49	**12**	22	0.344	49	15	19	0.329	49	18	16	0.467
**Dysplastic R. Olf. Bulb**	45	0	0	.	45	0	0	.	45	0	0	.
**Hypoplastic Brainstem**	46	0	0	.	46	0	0	.	46	0	0	.
**Dysplastic Brainstem**	47	**6**	12	0.814	47	10	8	0.100	47	11	7	0.196
**Hypoplastic Midbrain**	47	0	0	.	47	0	0	.	47	0	0	.
**Dysplastic Midbrain**	47	**7**	10	0.282	47	10	7	0.055	47	9	8	0.687
**BDS Dichotomized**	49	**14**	31	0.942	49	18	27	0.970	49	24	21	0.612
**BDS Hipp. or Cerebellum**	49	**14**	31	0.693	49	18	27	0.704	49	24	21	0.287

** Two Sample Student T-test used to examine incidence differences between groupings.*

Supplemental Table S7: Ohia Mouse Mutant Cohort Regional Cerebral Volumes Compared to Wild Type (WT)

			**Raw (Non-normalized) Volume**			**Normalized Volume**		
**Structure**	N Ohia	N WT	Ohia Mean (x10^8 μm^3)	WT Mean (x10^8 μm^3)	P value *	Ohia Percent TBV	WT Percent TBV	P value *
**Total Volume**	25	10	45.88	54.5	0.297	.	.	.
**Intraventricular Vol.**	11	9	**1.968**	0.531	**0.009**	**0.043**	0.011	**0.015**
**Supratentorial**	25	10	34.68	**68.46**	**0.037**	0.745	**1.287**	**0.01**
**Infratentorial**	25	10	10	11.81	0.358	0.232	0.212	0.208
**L. Hippocampus**	21	10	0.852	1.039	0.336	0.017	0.019	0.293
**R. Hippocampus**	20	10	0.831	0.981	0.431	0.016	0.018	0.406
**L. Olfactory Bulb**	18	10	2.017	2.212	0.59	0.038	0.041	0.121
**R. Olfactory Bulb**	17	10	2.066	2.188	0.711	0.038	0.041	0.057
**L. Subcortical**	25	10	4.104	**5.884**	**0.041**	0.097	0.107	0.223
**R. Subcortical**	25	10	4.073	**5.805**	**0.044**	0.096	0.106	0.178
**L. Cortex**	24	10	8.159	9.133	0.514	0.167	0.171	0.725
**R. Cortex**	23	10	8.192	9.392	0.445	0.162	0.174	0.295
**Cerebellum**	24	9	1.624	**2.451**	**0.005**	0.038	0.043	0.095
**Pons**	25	10	2.205	2.656	0.37	0.052	0.048	0.367
**Medulla**	24	10	6.459	6.803	0.77	**0.15**	0.122	**0.024**
**Hypothalamus**	7	10	0.118	0.148	0.267	0.002	0.003	0.074
**Choroid Plexus**	24	10	0.232	0.228	0.959	0.006	0.004	0.189
**Midbrain**	25	10	5.802	5.346	0.605	**0.138**	0.098	**0.005**

Supplemental Table S8A: Non-normalized Regional Cerebral Volumes of Ohia CHD cohorts: Comparison of Cardiac Lesion Subgroups

	**CHD vs. No-CHD**					**Single vs. Biventricular Morphology**				
	CHD		No CHD		P value *	Single		Biventricular		P value *
**Structure**	N	Mean (x10^8 μm^3)	N	Mean (x10^8 μm^3)		N	Mean (x10^8 μm^3)	N	Mean (x10^8 μm^3)	
**R. Hippocampus**	7	0.72	11	0.96	0.340	1	0.07	10	0.79	.
**R. Olf. Bulb**	6	1.93	9	2.36	0.500	0	.	9	1.93	.
**R. Subcortical**	7	3.59	16	4.97	0.237	2	2.45	14	**3.76**	**0.006**
**R. Cortex**	7	6.99	14	10.58	0.188	1	2.43	13	7.34	.
**Intraventricular Vol.**	2	2.10	8	1.70	0.535	1	2.29	7	2.07	.
**Midbrain**	7	5.60	16	6.53	0.597	2	4.67	14	5.73	0.192
**L. Hippocampus**	7	0.76	12	1.05	0.252	1	0.03	11	0.82	.
**L. Olf. Bulb**	6	1.79	10	2.52	0.314	0	.	10	1.79	.
**L. Subcortical**	7	3.65	16	5.00	0.242	2	2.79	14	**3.78**	**0.036**
**L. Cortex**	7	7.08	15	10.64	0.197	1	2.72	14	7.39	.
**Cerebellum**	7	1.61	15	1.61	1.000	1	1.00	14	1.65	.
**Pons**	7	2.08	16	2.50	0.572	2	1.20	14	**2.21**	**0.024**
**Medulla**	7	5.93	15	7.47	0.319	1	5.76	14	5.95	.
**Hypothalamus**	3	0.14	3	0.09	0.456	0	.	3	0.14	.
**Choroid Plexus**	6	0.26	16	0.21	0.530	2	0.32	14	0.25	0.743
**Supratentorial**	7	30.18	16	44.59	0.207	2	14.00	14	32.49	0.057
**Infratentorial**	7	9.18	16	11.62	0.364	2	4.58	14	9.83	0.374
**Total Volume**	7	40.65	16	57.36	0.230	2	21.18	14	43.43	0.247

** Welch–Satterthwaite t-test was used to test volume differences among individual structures between groups.*

Supplemental Table S8B: Non-normalized Regional Cerebral Volumes of Ohia CHD cohorts: Comparison of Cardiac Lesion Subgroups

	**Conotruncal vs. Non-Conotruncal**					**Cyanotic vs. Acyanotic Lesion**					**Arch Obstruction vs. No Arch Obstruction**				
	Conotruncal		Non Conotruncal		P value *	Cyanotic		Acyanotic		P value *	Arch Obstruction		No Obstruction		P value *
**Structure**	N	Mean (x10^{8} μm^{3})	N	Mean (x10^{8} μm^{3})		N	Mean (x10^{8} μm^{3})	N	Mean (x10^{8} μm^{3})		N	Mean (x10^{8} μm^{3})	N	Mean (x10^{8} μm^{3})	
**R. Hippocampus**	3	0.49	9	0.79	0.117	5	0.53	6	0.89	0.114	4	0.80	7	0.68	0.513
**R. Olf. Bulb**	2	1.47	8	1.94	0.159	3	1.51	6	2.14	0.121	4	1.66	5	2.14	0.325
**R. Subcortical**	4	2.87	13	**3.77**	**0.043**	7	2.96	9	4.08	0.057	7	3.54	9	3.63	0.889
**R. Cortex**	3	5.88	12	7.26	0.412	5	5.57	9	7.78	0.249	6	6.03	8	7.71	0.419
**Intraventricular Vol.**	2	2.10	6	2.10	0.999	3	2.16	5	2.06	0.944	3	1.69	5	2.34	0.643
**Midbrain**	4	5.22	13	5.61	0.675	7	5.02	9	6.05	0.398	7	4.62	9	6.36	0.159
**L. Hippocampus**	4	0.59	9	0.82	0.228	6	0.55	6	**0.97**	**0.044**	4	0.81	8	0.73	0.679
**L. Olf. Bulb**	3	1.60	8	1.79	0.556	4	1.56	6	1.95	0.340	4	1.39	6	2.06	0.097
**L. Subcortical**	4	3.11	13	3.78	0.141	7	3.22	9	3.99	0.211	7	3.40	9	3.85	0.496
**L. Cortex**	4	6.43	12	7.30	0.591	6	6.22	9	7.66	0.434	6	5.98	9	7.81	0.334
**Cerebellum**	4	1.56	12	1.61	0.811	6	1.54	9	1.65	0.657	6	1.45	9	1.71	0.313
**Pons**	4	1.99	13	2.07	0.788	7	1.89	9	2.23	0.429	7	1.65	9	2.42	0.073
**Medulla**	4	5.69	12	6.13	0.493	6	6.33	9	5.67	0.473	6	5.87	9	5.98	0.913
**Hypothalamus**	1	0.11	3	0.12	.	2	0.09	1	0.23	.	0	.	3	0.14	.
**Choroid Plexus**	4	0.24	13	0.26	0.878	7	0.25	9	0.26	0.936	7	0.31	9	0.22	0.579
**Supratentorial**	4	26.22	13	31.25	0.344	7	24.06	9	34.93	0.138	7	25.55	9	33.77	0.285
**Infratentorial**	4	9.27	13	9.24	0.98	7	8.66	9	9.58	0.610	7	7.92	9	10.16	0.213
**Total Volume**	4	35.58	13	42.06	0.314	7	33.52	9	46.19	0.163	7	34.31	9	45.57	0.238

** Welch–Satterthwaite t-test was used to test volume differences among individual structures between groups.*

Supplemental Table S9A: Normalized Regional Cerebral Volumes of Ohia CHD cohorts: Comparison of Cardiac Lesion Subgroups

	**CHD vs. No-CHD**			**Single vs.Biventricular Morphology**		
**Structure**	CHD Percent TBV	No CHD Percent TBV	P value *	Single Percent TBV	Bivent. Percent TBV	P value *
**R. Hippocampus**	0.015	0.017	0.376	0.002	0.016	.
**R. Olf. Bulb**	0.037	0.039	0.709	.	0.037	.
**R. Subcortical**	0.099	0.086	0.198	0.150	0.092	0.5485
**R. Cortex**	**0.150**	0.183	**0.026**	0.076	0.156	.
**Intraventricular Vol.**	0.049	0.026	0.155	0.072	0.046	.
**Midbrain**	**0.155**	0.111	**0.044**	0.289	0.136	0.4591
**L. Hippocampus**	0.016	0.019	0.262	0.001	0.018	.
**L. Olf. Bulb**	0.037	0.040	0.521	.	0.037	.
**L. Subcortical**	0.102	0.087	0.206	0.171	0.092	0.4917
**L. Cortex**	0.159	0.184	0.106	0.085	0.164	.
**Cerebellum**	**0.041**	0.030	**0.044**	0.031	0.042	.
**Pons**	0.055	0.046	0.292	0.070	0.053	0.6264
**Medulla**	0.155	0.141	0.516	0.180	0.153	.
**Hypothalamus**	0.002	0.002	0.611	.	0.002	.
**Choroid Plexus**	0.007	0.004	0.057	**0.015**	0.006	**0.0003**
**Supratentorial**	0.735	0.764	0.324	0.743	0.733	0.963
**Infratentorial**	0.239	0.218	0.506	0.176	0.248	0.5293

** Welch–Satterthwaite t-test was used to test percent volume differences among individual structures between groups.*

Supplemental Table S9B: Normalized Regional Cerebral Volumes of Ohia CHD cohorts: Comparison of Cardiac Lesion Subgroups

	**Conotruncal vs. Non-Conotruncal**			**Cyanotic vs. Acyanotic Lesion**			**Arch Obstruction vs. No Arch Obstruction**		
**Structure**	Cono. Percent TBV	Non Cono. Percent TBV	P value *	Cyanotic Percent TBV	Acyanotic Percent TBV	P value *	Obstr. Percent TBV	No Obstr. Percent TBV	P value *
**R. Hippocampus**	0.013	0.016	0.412	0.013	0.017	0.293	0.019	0.013	0.097
**R. Olf. Bulb**	0.037	0.036	0.820	0.035	0.038	0.329	0.037	0.037	0.937
**R. Subcortical**	0.083	0.103	0.217	0.103	0.096	0.783	0.121	0.082	0.086
**R. Cortex**	0.155	0.151	0.890	0.139	0.156	0.517	0.145	0.154	0.760
**Intraventricular Vol.**	0.064	0.044	0.809	0.067	0.039	0.532	0.036	0.057	0.470
**Midbrain**	0.146	0.154	0.756	0.180	0.135	0.328	0.171	0.142	0.529
**L. Hippocampus**	0.017	0.016	0.876	0.014	0.018	0.377	0.019	0.015	0.242
**L. Olf. Bulb**	0.044	0.034	0.079	0.040	0.035	0.344	0.032	0.040	0.075
**L. Subcortical**	0.089	0.104	0.413	0.113	0.093	0.440	0.120	0.087	0.211
**L. Cortex**	0.183	0.153	0.477	0.166	0.155	0.739	0.144	0.169	0.402
**Cerebellum**	0.044	0.040	0.347	0.041	0.041	0.978	0.044	0.039	0.624
**Pons**	0.056	0.054	0.643	0.060	0.051	0.241	0.056	0.054	0.821
**Medulla**	0.162	0.155	0.735	0.170	0.145	0.289	0.166	0.147	0.498
**Hypothalamus**	0.003	0.002	.	0.002	0.003	.	.	0.002	.
**Choroid Plexus**	0.007	0.007	0.919	0.009	0.006	0.510	0.009	0.006	0.374
**Supratentorial**	0.734	0.734	0.997	0.734	0.735	0.969	0.746	0.726	0.665
**Infratentorial**	0.263	0.234	0.278	0.241	0.237	0.900	0.236	0.241	0.907

** Welch–Satterthwaite t-test was used to test percent volume differences among individual structures between groups.*

Supplemental Table S10: OVERVIEW OF OHIA MUTANT MODEL GENOTYPE GROUPS STUDIES: Congenital heart disease lesions and Holoprosencephaly Incidence

	A†	B‡	C§	D‖	E¶	F#
**N Total**	24	8	7	4	0	20
**CHD**	21	7	6	3	0	10
**Holoprosencephaly**	19	6	4	4	0	8
**Conotruncal**	7	3	0	1	0	5
**Single Ventricle**	1	1	0	0	0	0
**Cyanotic**	8	4	1	1	0	6
**Arch Obstruction**	16	1	3	2	0	2

†Group A: N 24, Genotype: Pcdha9(m/m) Sap130(m/m)

‡ Group B: N 8, Genotype: Pcdha9(m/+) Sap130(m/m)

§ Group C: N 7, Genotype: Pcdha9(+/+) Sap130(m/m)

|| Group D: N 4, Genotype: Pcdha9(m/m) Sap130(m/+)

¶ Group E: N 0, Genotype: Pcdha9(m/m) Sap130(+/+)

Group F: N 27, Genotype: Pcdha9(m/+) or (+/+) Sap130(m/+) or (+/+)

Supplemental Table S11A: Genotype/Brain Dysplasia Score : Sap130(m/m), Pcdha9 (*/*) vs WT; Sap130(m/m), Pcdha9 (*/*) vs Sap130(*/+), Pcdha9 (*/+)

	ABC vs. WT Sap130(m/m), Pcdha9 (*/*) vs WT			ABC vs. F Sap130(m/m), Pcdha9 (*/*) vs Sap130(*/+), Pcdha9 (*/+)		
Structure	ABC Incidence	WT Incidence	P value *	ABC Incidence	F Incidence	P value *
**Aplastic Hippocampus**	05/39 (12.82%)	0/10 (0.00%)	0.232	05/39 (12.82%)	01/20 (5.00%)	0.347
**Hypoplastic Hippocampus**	06/38 (15.79%)	0/10 (0.00%)	0.179	06/38 (15.79%)	05/20 (25.00%)	0.395
**Combination Hippocampus**	11/39 (28.21%)	0/10 (0.00%)	0.057	11/39 (28.21%)	06/20 (30.00%)	0.885
**Dysplastic Hippocampus**	**27/38 (71.05%)**	0/10 (0.00%)	**<.001**	27/38 (71.05%)	12/20 (60.00%)	0.394
**Hypoplastic Cerebrum**	**12/38 (31.58%)**	0/10 (0.00%)	**0.040**	**12/38 (31.58%)**	01/20 (5.00%)	**0.021**
**Dysplastic Cerebrum**	**29/38 (76.32%)**	0/10 (0.00%)	**<.001**	**29/38 (76.32%)**	08/20 (40.00%)	**0.016**
**Aplastic Cerebellum**	01/39 (2.56%)	0/10 (0.00%)	0.609	01/39 (2.56%)	01/20 (5.00%)	0.625
**Hypoplastic Cerebellum**	04/37 (10.81%)	0/10 (0.00%)	0.277	04/37 (10.81%)	05/20 (25.00%)	0.161
**Combination Cerebellum**	05/39 (12.82%)	0/10 (0.00%)	0.232	05/39 (12.82%)	06/20 (30.00%)	0.109
**Dysplastic Cerebellum**	**34/37 (91.89%)**	0/10 (0.00%)	**<.001**	**34/37 (91.89%)**	09/20 (45.00%)	**<.001**
**Aplastic L. Olf. Bulb**	**23/39 (58.97%)**	0/10 (0.00%)	**0.001**	**23/39 (58.97%)**	06/20 (30.00%)	**0.035**
**Hypoplastic L. Olf. Bulb**	06/37 (16.22%)	0/10 (0.00%)	0.173	06/37 (16.22%)	02/20 (10.00%)	0.519
**Combination L. Olf. Bulb**	**29/39 (74.36%)**	0/10 (0.00%)	**<.001**	**29/39 (74.36%)**	08/20 (40.00%)	**0.010**
**Dysplastic L. Olf. Bulb**	01/37 (2.70%)	0/10 (0.00%)	0.599	01/37 (2.70%)	01/20 (5.00%)	0.653
**Aplastic R. Olf. Bulb**	**24/39 (61.54%)**	0/10 (0.00%)	**0.001**	**24/39 (61.54%)**	06/20 (30.00%)	**0.022**
**Hypoplastic R. Olf. Bulb**	04/36 (11.11%)	0/10 (0.00%)	0.270	04/36 (11.11%)	02/19 (10.53%)	0.947
**Combination R. Olf. Bulb**	**28/39 (71.79%)**	0/10 (0.00%)	**<.001**	**28/39 (71.79%)**	08/20 (40.00%)	**0.018**
**Dysplastic R. Olf. Bulb**	00/36 (0.00%)	0/10 (0.00%)	.	00/36 (0.00%)	00/19 (0.00%)	.
**Hypoplastic Brainstem**	00/37 (0.00%)	0/10 (0.00%)	.	00/37 (0.00%)	00/19 (0.00%)	.
**Dysplastic Brainstem**	**12/37 (32.43%)**	0/10 (0.00%)	**0.037**	12/37 (32.43%)	06/20 (30.00%)	0.850
**Hypoplastic Midbrain**	00/37 (0.00%)	0/10 (0.00%)	.	00/37 (0.00%)	00/20 (0.00%)	.
**Dysplastic Midbrain**	10/37 (27.03%)	0/10 (0.00%)	0.064	10/37 (27.03%)	06/20 (30.00%)	0.812
**BDS Dichotomized**	**35/39 (89.74%)**	0/10 (0.00%)	**<.001**	**35/39 (89.74%)**	13/20 (65.00%)	**0.021**

** Chi-square analysis was used to compare individual structures and BDS Dichotomized.*

Supplemental Table S11B: Genotype/Brain Dysplasia Score: Sap130(m/m), Pcdha9 (m/*) vs Sap130(*/+), Pcdha9 (*/+); Sap130(m/m), Pcdha9 (m/m) vs Sap130(m/m), Pcdha9 (+/+)

	AB vs. F Sap130(m/m), Pcdha9 (m/*) vs Sap130(*/+), Pcdha9 (*/+)			A vs. C Sap130(m/m), Pcdha9 (m/m) vs Sap130(m/m), Pcdha9 (+/+)		
Structure	AB Incidence	F Incidence	P value *	A Incidence	C Incidence	P value *
**Aplastic Hippocampus**	05/32 (15.63%)	01/20 (5.00%)	0.243	05/24 (20.83%)	00/7 (0.00%)	0.187
**Hypoplastic Hippocampus**	06/31 (19.35%)	05/20 (25.00%)	0.632	04/23 (17.39%)	00/7 (0.00%)	0.236
**Combination Hippocampus**	11/32 (34.38%)	06/20 (30.00%)	0.744	09/24 (37.50%)	00/7 (0.00%)	0.054
**Dysplastic Hippocampus**	21/31 (67.74%)	12/20 (60.00%)	0.572	15/23 (65.22%)	06/7 (85.71%)	0.300
**Hypoplastic Cerebrum**	**09/31 (29.03%)**	01/20 (5.00%)	**0.035**	07/23 (30.43%)	03/7 (42.86%)	0.542
**Dysplastic Cerebrum**	**24/31 (77.42%)**	08/20 (40.00%)	**0.019**	18/23 (78.26%)	05/7 (71.43%)	0.708
**Aplastic Cerebellum**	01/32 (3.13%)	01/20 (5.00%)	0.732	01/24 (4.17%)	00/7 (0.00%)	0.583
**Hypoplastic Cerebellum**	04/30 (13.33%)	05/20 (25.00%)	0.293	02/22 (9.09%)	00/7 (0.00%)	0.408
**Combination Cerebellum**	05/32 (15.63%)	06/20 (30.00%)	0.217	03/24 (12.50%)	00/7 (0.00%)	0.325
**Dysplastic Cerebellum**	**27/30 (90.00%)**	09/20 (45.00%)	**0.001**	20/22 (90.91%)	07/7 (100.00%)	0.408
**Aplastic L. Olf. Bulb**	**19/32 (59.38%)**	06/20 (30.00%)	**0.039**	14/24 (58.33%)	04/7 (57.14%)	0.955
**Hypoplastic L. Olf. Bulb**	04/30 (13.33%)	02/20 (10.00%)	0.722	03/22 (13.64%)	02/7 (28.57%)	0.362
**Combination L. Olf. Bulb**	**23/32 (71.88%)**	08/20 (40.00%)	**0.023**	17/24 (70.83%)	06/7 (85.71%)	0.429
**Dysplastic L. Olf. Bulb**	01/30 (3.33%)	01/20 (5.00%)	0.768	01/22 (4.55%)	00/7 (0.00%)	0.566
**Aplastic R. Olf. Bulb**	**20/32 (62.50%)**	06/20 (30.00%)	**0.023**	15/24 (62.50%)	04/7 (57.14%)	0.798
**Hypoplastic R. Olf. Bulb**	03/29 (10.34%)	02/19 (10.53%)	0.984	02/21 (9.52%)	01/7 (14.29%)	0.724
**Combination R. Olf. Bulb**	**23/32 (71.88%)**	08/20 (40.00%)	**0.023**	17/24 (70.83%)	05/7 (71.43%)	0.976
**Dysplastic R. Olf. Bulb**	00/29 (0.00%)	00/19 (0.00%)	.	00/21 (0.00%)	00/7 (0.00%)	.
**Hypoplastic Brainstem**	00/30 (0.00%)	00/19 (0.00%)	.	00/22 (0.00%)	00/7 (0.00%)	.
**Dysplastic Brainstem**	12/30 (40.00%)	06/20 (30.00%)	0.471	**10/22 (45.45%)**	00/7 (0.00%)	**0.028**
**Hypoplastic Midbrain**	00/30 (0.00%)	00/20 (0.00%)	.	00/22 (0.00%)	00/7 (0.00%)	.
**Dysplastic Midbrain**	10/30 (33.33%)	06/20 (30.00%)	0.805	**09/22 (40.91%)**	00/7 (0.00%)	**0.042**
**BDS Dichotomized**	28/32 (87.50%)	13/20 (65.00%)	0.053	21/24 (87.50%)	07/7 (100.00%)	0.325

** Chi-square analysis was used to compare individual structures and BDS Dichotomized*

Supplemental Table S11C: Genotype/Brain Dysplasia Score: Sap130(m/m), Pcdha9 (m/+) vs Sap130(m/+), Pcdha9 (m/m)

	**B vs. D**		
	Sap130(m/m), Pcdha9 (m/+) vs Sap130(m/+), Pcdha9 (m/m)		
**Structure**	B Incidence	D Incidence	P value *
**Aplastic Hippocampus**	00/8 (0.00%)	**02/4 (50.00%)**	**0.029**
**Hypoplastic Hippocampus**	02/8 (25.00%)	02/4 (50.00%)	0.387
**Combination Hippocampus**	02/8 (25.00%)	**04/4 (100.00%)**	**0.014**
**Dysplastic Hippocampus**	06/8 (75.00%)	02/4 (50.00%)	0.387
**Hypoplastic Cerebrum**	02/8 (25.00%)	00/4 (0.00%)	0.273
**Dysplastic Cerebrum**	06/8 (75.00%)	04/4 (100.00%)	0.273
**Aplastic Cerebellum**	00/8 (0.00%)	00/4 (0.00%)	.
**Hypoplastic Cerebellum**	02/8 (25.00%)	00/4 (0.00%)	0.273
**Combination Cerebellum**	02/8 (25.00%)	00/4 (0.00%)	0.273
**Dysplastic Cerebellum**	07/8 (87.50%)	04/4 (100.00%)	0.460
**Aplastic L. Olf. Bulb**	05/8 (62.50%)	03/4 (75.00%)	0.665
**Hypoplastic L. Olf. Bulb**	01/8 (12.50%)	01/4 (25.00%)	0.584
**Combination L. Olf. Bulb**	06/8 (75.00%)	04/4 (100.00%)	0.273
**Dysplastic L. Olf. Bulb**	00/8 (0.00%)	00/4 (0.00%)	.
**Aplastic R. Olf. Bulb**	05/8 (62.50%)	03/4 (75.00%)	0.665
**Hypoplastic R. Olf. Bulb**	01/8 (12.50%)	01/4 (25.00%)	0.584
**Combination R. Olf. Bulb**	06/8 (75.00%)	04/4 (100.00%)	0.273
**Dysplastic R. Olf. Bulb**	00/8 (0.00%)	00/4 (0.00%)	.
**Hypoplastic Brainstem**	00/8 (0.00%)	00/4 (0.00%)	.
**Dysplastic Brainstem**	02/8 (25.00%)	02/4 (50.00%)	0.387
**Hypoplastic Midbrain**	00/8 (0.00%)	00/4 (0.00%)	.
**Dysplastic Midbrain**	01/8 (12.50%)	02/4 (50.00%)	0.157
**BDS Dichotomized**	07/8 (87.50%)	04/4 (100.00%)	0.460

** Welch–Satterthwaite t-test was used to test volume differences among individual structures between groups.*

Supplemental Table S12A: Genotype/Regional Brain Volumes: Sap130(m/m), Pcdha9 (*/*) vs WT; Sap130(m/m), Pcdha9 (*/*) vs Sap130(*/+), Pcdha9 (*/+)

	**ABC vs. WT**					**ABC vs. F**				
	Sap130(m/m), Pcdha9 (*/*) vs WT					Sap130(m/m), Pcdha9 (*/*) vs Sap130(*/+), Pcdha9 (*/+)				
	ABC		WT		P value *	ABC		F		P value *
**Structure**	N	Mean (x10^8 μm^3)	N	Mean (x10^8 μm^3)		N	Mean (x10^8 μm^3)	N	Mean (x10^8 μm^3)	
**R. Hippocampus**	9	0.61	10	0.98	0.077	9	0.61	9	**1.03**	**0.041**
**R. Olf. Bulb**	7	1.63	10	2.19	0.171	7	1.63	8	2.50	0.096
**R. Subcortical**	14	3.31	10	**5.80**	**0.006**	14	3.31	9	**5.06**	**0.050**
**R. Cortex**	12	6.00	10	**9.39**	**0.047**	12	6.00	9	**11.26**	**0.016**
**Intraventricular Vol.**	7	1.81	9	0.53	0.083	7	1.81	3	2.50	0.530
**Midbrain**	14	4.70	10	5.35	0.397	14	4.70	9	7.57	0.078
**L. Hippocampus**	10	0.63	10	1.04	0.051	10	0.63	9	**1.09**	**0.031**
**L. Olf. Bulb**	8	1.52	10	2.21	0.085	8	1.52	8	**2.60**	**0.048**
**L. Subcortical**	14	3.39	10	**5.88**	**0.007**	14	3.39	9	5.04	0.062
**L. Cortex**	13	6.15	10	9.13	0.061	13	6.15	9	**11.13**	**0.022**
**Cerebellum**	13	1.53	9	**2.45**	**0.003**	13	1.53	9	1.74	0.516
**Pons**	14	1.84	10	2.66	0.106	14	1.84	9	2.74	0.144
**Medulla**	13	5.82	10	6.80	0.414	13	5.82	9	7.13	0.291
**Hypothalamus**	1	0.07	10	0.15	.	1	0.07	5	0.12	.
**Choroid Plexus**	14	0.27	10	0.23	0.647	14	0.27	8	0.18	0.272
**Supratentorial**	14	25.95	10	**68.46**	**0.013**	14	25.95	9	**47.72**	**0.018**
**Infratentorial**	14	8.66	10	11.81	0.128	14	8.66	9	11.68	0.149
**Total Volume**	14	35.49	10	**54.50**	**0.028**	14	35.49	9	**61.23**	**0.022**

** Welch–Satterthwaite t-test was used to test volume differences among individual structures between groups.*

Supplemental Table S12B: Genotype/Regional Brain Volumes: Sap130(m/m), Pcdha9 (m/*) vs Sap130(*/+), Pcdha9 (*/+);Sap130(m/m), Pcdha9 (m/m) vs Sap130(m/m), Pcdha9 (+/+)

	**AB vs. F**					**A vs. C**				
	Sap130(m/m), Pcdha9 (m/*) vs Sap130(*/+), Pcdha9 (*/+)					Sap130(m/m), Pcdha9 (m/m) vs Sap130(m/m), Pcdha9 (+/+)				
	AB		F		P value *	A		C		P value *
**Structure**	N	Mean (x10^8 μm^3)	N	Mean (x10^8 μm^3)		N	Mean (x10^8 μm^3)	N	Mean (x10^8 μm^3)	
**R. Hippocampus**	7	0.52	9	**1.03**	**0.021**	3	0.56	2	0.90	0.281
**R. Olf. Bulb**	5	1.54	8	2.50	0.121	3	1.16	2	1.87	0.195
**R. Subcortical**	10	2.94	9	**5.06**	**0.023**	5	2.27	4	**4.24**	**0.022**
**R. Cortex**	8	5.79	9	**11.26**	**0.029**	4	4.72	4	6.41	0.423
**Intraventricular Vol.**	4	1.64	3	2.50	0.532	2	0.05	3	2.04	0.128
**Midbrain**	10	4.48	9	7.57	0.064	5	3.88	4	5.26	0.193
**L. Hippocampus**	8	0.58	9	**1.09**	**0.024**	3	0.56	2	0.84	0.200
**L. Olf. Bulb**	6	1.54	8	2.60	0.080	3	1.07	2	1.45	0.309
**L. Subcortical**	10	3.10	9	**5.04**	**0.038**	5	2.34	4	**4.11**	**0.034**
**L. Cortex**	9	5.93	9	**11.13**	**0.029**	4	4.58	4	6.63	0.327
**Cerebellum**	9	1.49	9	1.74	0.491	4	1.26	4	1.61	0.302
**Pons**	10	1.77	9	2.74	0.126	5	1.45	4	2.01	0.254
**Medulla**	9	5.11	9	7.13	0.109	4	4.44	4	7.43	0.052
**Hypothalamus**	0	.	5	0.12	.	0	.	1	0.07	.
**Choroid Plexus**	10	0.22	8	0.18	0.532	5	0.13	4	0.41	0.327
**Supratentorial**	10	23.88	9	**47.72**	**0.014**	5	18.08	4	31.12	0.089
**Infratentorial**	10	7.70	9	11.68	0.072	5	6.02	4	**11.07**	**0.047**
**Total Volume**	10	32.15	9	**61.23**	**0.014**	5	24.09	4	**43.84**	**0.049**

** Welch–Satterthwaite t-test was used to test volume differences among individual structures between groups.*

Supplemental Table S13A: Genotype/Regional Brain Volume: Sap130(m/m), Pcdha9 (*/*) vs WT; Sap130(m/m), Pcdha9 (*/*) vs Sap130(*/+), Pcdha9 (*/+)

	**ABC vs. WT**			**ABC vs. F**		
	Sap130(m/m), Pcdha9 (*/*) vs WT			Sap130(m/m), Pcdha9 (*/*) vs Sap130(*/+), Pcdha9 (*/+)		
**Structure**	ABC Percent TBV	WT Percent TBV	P value *	ABC Percent TBV	F Percent TBV	P value *
**R. Hippocampus**	0.015	0.018	0.238	0.015	0.017	0.328
**R. Olf. Bulb**	0.036	**0.041**	**0.037**	0.036	0.039	0.287
**R. Subcortical**	0.103	0.106	0.792	0.103	0.083	0.089
**R. Cortex**	0.147	0.174	0.105	0.147	**0.185**	**0.028**
**Intraventricular Vol.**	0.049	0.011	0.063	0.049	0.034	0.420
**Midbrain**	**0.153**	0.098	**0.027**	0.153	0.121	0.191
**L. Hippocampus**	0.016	0.019	0.304	0.016	0.018	0.432
**L. Olf. Bulb**	0.036	0.041	0.117	0.036	0.041	0.202
**L. Subcortical**	0.106	0.107	0.898	0.106	0.083	0.086
**L. Cortex**	0.157	0.171	0.430	0.157	0.183	0.138
**Cerebellum**	0.043	0.043	0.864	**0.043**	0.030	**0.010**
**Pons**	0.056	0.048	0.102	0.056	0.045	0.104
**Medulla**	**0.166**	0.122	**0.005**	**0.166**	0.125	**0.043**
**Hypothalamus**	0.001	0.003	.	0.001	0.002	.
**Choroid Plexus**	0.008	0.004	0.052	**0.008**	0.003	**0.012**
**Supratentorial**	0.726	**1.287**	**0.009**	0.726	0.774	0.092
**Infratentorial**	0.251	0.212	0.075	0.251	0.201	0.083

** Welch–Satterthwaite t-test was used to test percent volume differences among individual structures between groups.*

Supplemental Table S13B: Genotype/Regional Brain Volume: Sap130(m/m), Pcdha9 (m/*) vs Sap130(*/+), Pcdha9 (*/+);Sap130(m/m), Pcdha9 (m/m) vs Sap130(m/m), Pcdha9 (+/+)

	**AB vs. F**			**A vs. C**		
	Sap130(m/m), Pcdha9 (m/*) vs Sap130(*/+), Pcdha9 (*/+)			Sap130(m/m), Pcdha9 (m/m) vs Sap130(m/m), Pcdha9 (+/+)		
**Structure**	AB Percent TBV	F Percent TBV	P value *	A Percent TBV	C Percent TBV	P value *
**R. Hippocampus**	0.014	0.017	0.256	0.017	0.018	0.963
**R. Olf. Bulb**	0.036	0.039	0.304	0.035	0.036	0.830
**R. Subcortical**	0.105	0.083	0.168	0.119	0.097	0.486
**R. Cortex**	0.148	0.185	0.132	0.150	0.147	0.940
**Intraventricular Vol.**	0.052	0.034	0.599	0.004	0.046	0.057
**Midbrain**	0.164	0.121	0.195	0.195	0.127	0.324
**L. Hippocampus**	0.016	0.018	0.505	0.018	0.016	0.756
**L. Olf. Bulb**	0.038	0.041	0.529	0.032	0.028	0.344
**L. Subcortical**	0.111	0.083	0.135	0.125	0.093	0.407
**L. Cortex**	0.159	0.183	0.334	0.147	0.152	0.900
**Extra-axial CSF**	0.087	0.075	0.901	.	0.050	.
**Cerebellum**	**0.046**	0.030	**0.012**	0.052	0.038	0.256
**Pons**	**0.060**	0.045	**0.049**	0.067	0.048	0.164
**Medulla**	0.165	0.125	0.091	0.184	0.169	0.679
**Hypothalamus**	.	0.002	.	.	0.001	.
**Choroid Plexus**	**0.008**	0.003	**0.029**	0.007	0.009	0.778
**Supratentorial**	0.733	0.774	.	0.745	0.707	0.533
**Infratentorial**	0.250	0.201	0.256	0.255	0.255	0.995

** Welch–Satterthwaite t-test was used to test percent volume differences among individual structures between groups.*

Supplemental Figures

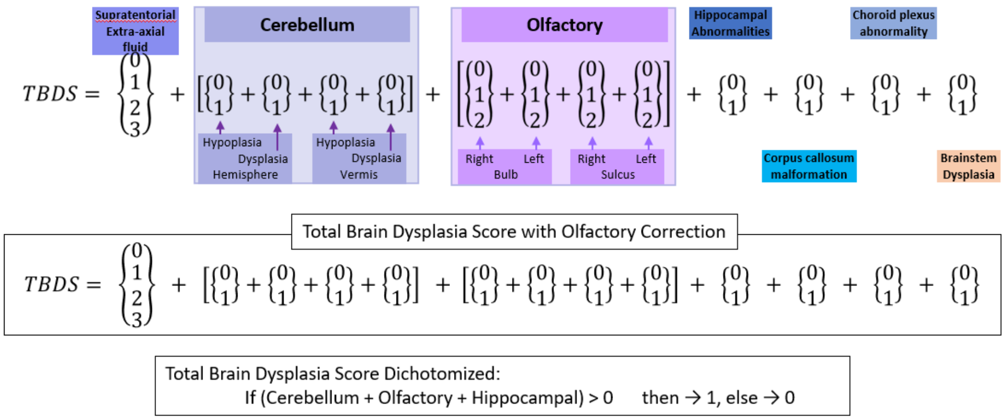

Supplemental Figure S1. Components of Human Brain Dysplasia Scoring Criteria. Our brain dysplasia scoring criteria consisted of 13 distinct observations. Supratentorial extra-axial fluid was assessed for normality, cerebellar hemisphere and vermis were assessed for hypoplasia and dysplasia, right and left olfactory bulb and olfactory sulcus were examined for any abnormalities. Additionally, the hippocampus, corpus collosum, choroid plexus, and brainstem were checked for abnormalities.

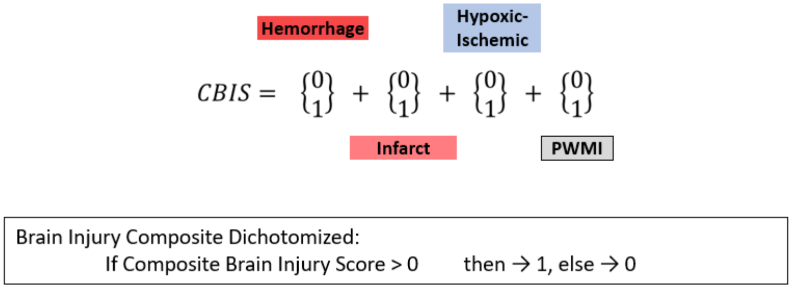

Supplemental Figure S2. Components of Composite Brain Injury Score. Brain injury score was composed of four measures: hemorrhage, infarct, hypoxic ischemia, and perinatal white matter injury. Brain injury score was binary, if any injury was present in the above measures, the subject was considered to have a brain injury.

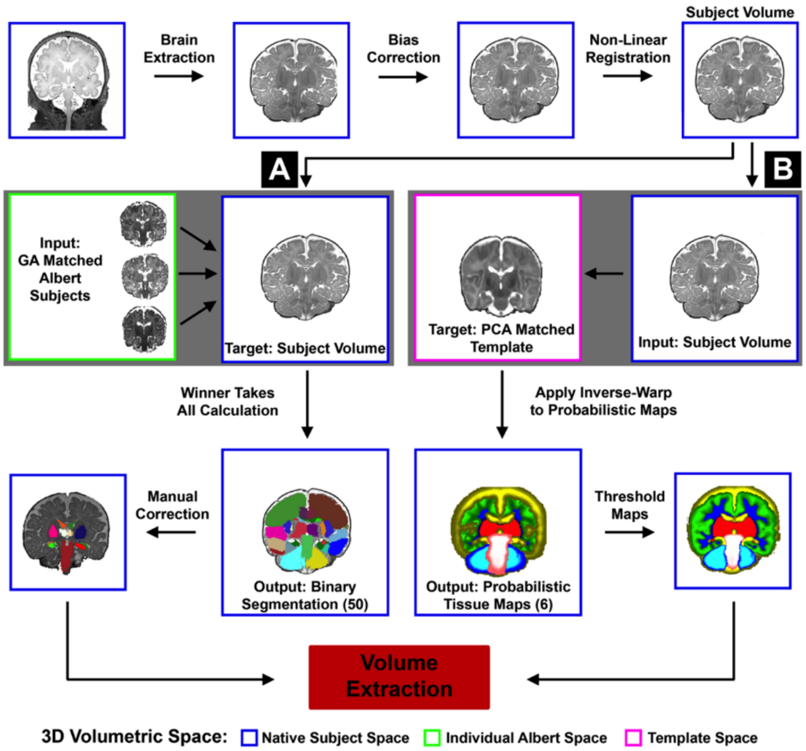

Supplemental Figure S3. Flow Diagram for NeBSS, a Semi-automated Neonatal Segmentation Pipeline. Briefly, subject MR images are input into NeBSS which has two branches. Branch A uses the Albert Neonatal Atlas and outputs 50 distinct volumetric brain structures in the subject space. Branch B uses the Serag Neonatal Brain Atlas probability maps to output 10 volumetric brain regions.

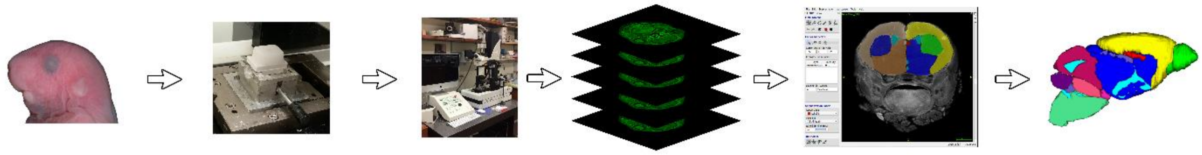

Supplemental Figure S4. Flow Diagram of Sample Preparation, ECM Analysis, and Volumetric Analysis. Samples selected for analysis were first necropsied. Post-necropsy samples were dehydrated using a Sakura Tissue Tek system and processed on the ECM machine. Samples were then manually segmented using ITK-Snap before volumetric results were analyzed.

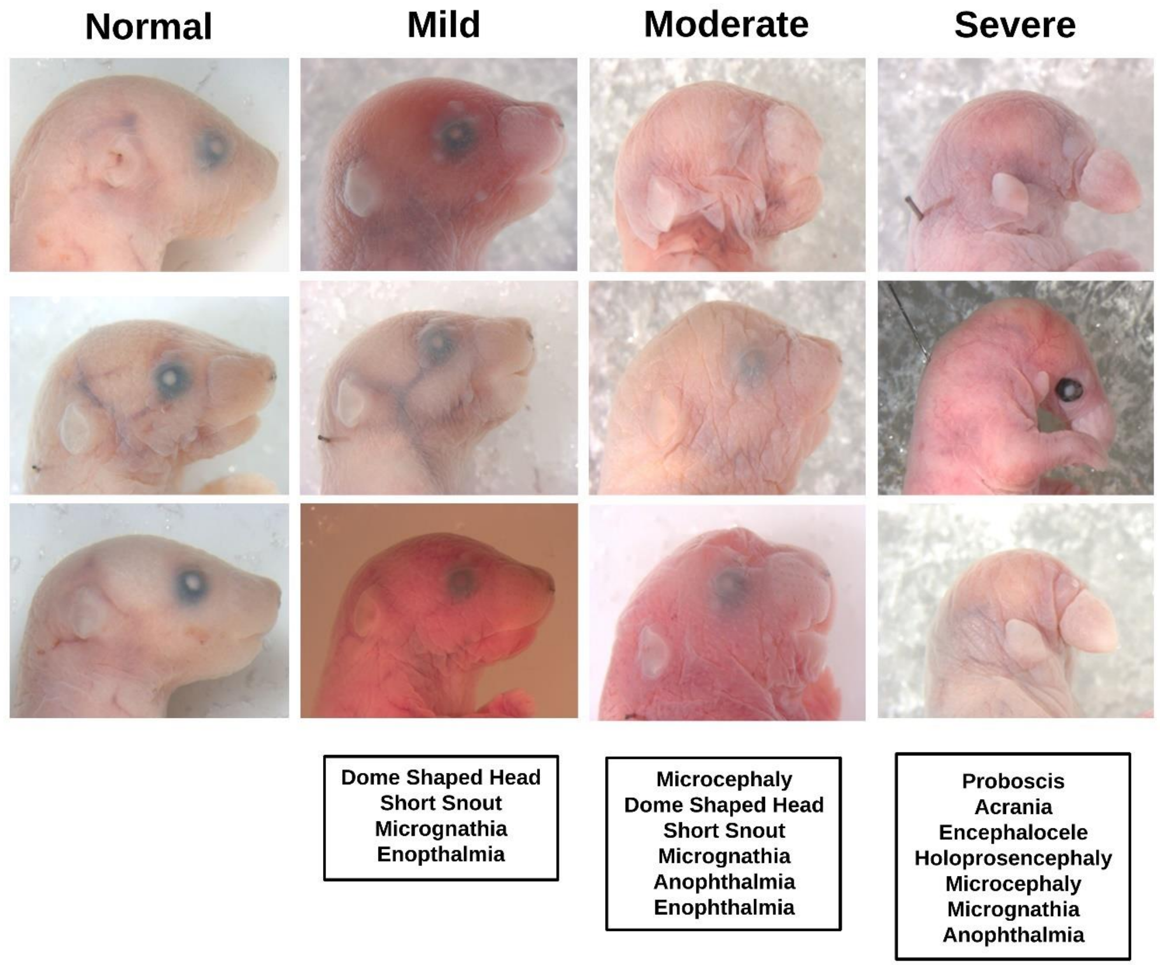

Supplemental Figure S5. Necropsy Among Mutant Mice Showed Variety and Differing Degree of Severity of Malformations. Mice in the mouse screen presented with craniofacial anomalies with varying degrees of severity. More mild forms of errant craniofacial anatomy presented with mild defects in the ears and eyes. Moderate forms presented as more severe ear and eye defects as well as a shortened snout. Lastly, severe defects presented with large structural defects in the craniofacial anatomy, often with underlying structural brain defects as well.

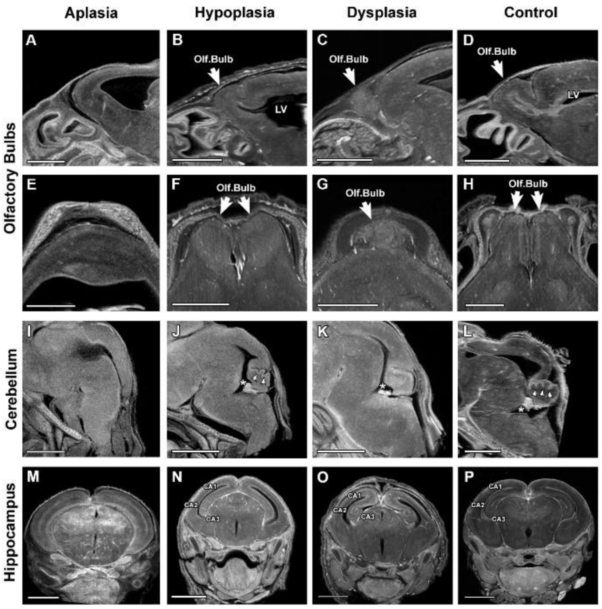

Supplemental Figure S6. Individual Structures Showed a Range of Abnormality in Screen Mice. Episcopic Fluorescence Image Capture (ECM) was carried out on prepared mouse brains. Samples presented with varying degrees of abnormalities. Three major structures analyzed were olfactory bulbs, cerebellum, and hippocampus. Within each structure, mice had a variety of defects including aplasia, hypoplasia, and dysplasia.

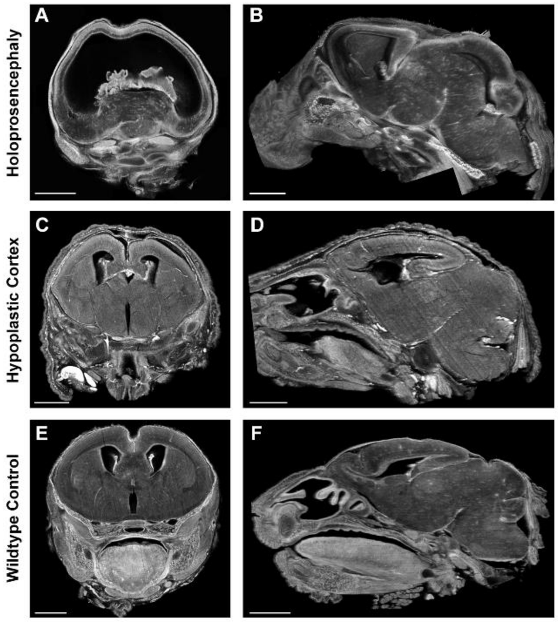

Supplemental Figure S7. Varying Degree of Cerebral Abnormalities Present in Screen Mice. ECM analysis found that mice had varying degrees of severity of cerebral abnormalities including holoprosencephaly and cerebral hypoplasia.

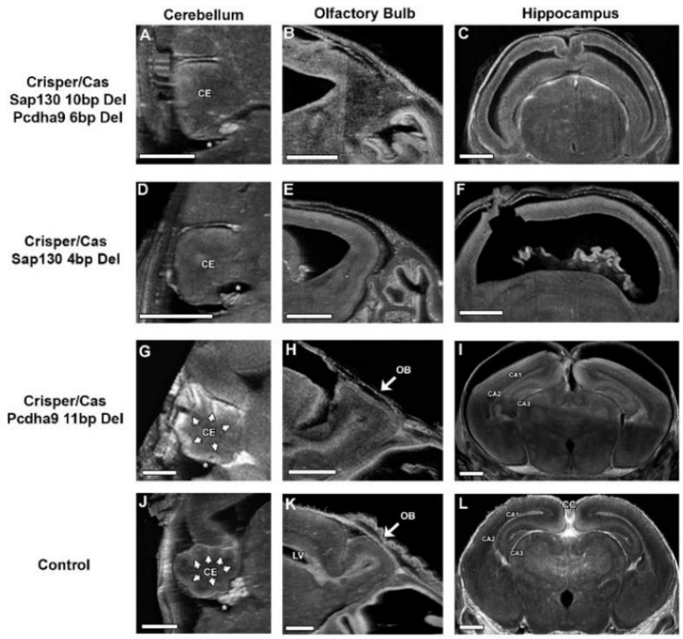

Supplemental Figure S8. Crispr/Cas Figure Mutant Animals Showed Similar Phenotype to Screen Mice. Using ECM, Crispr/Cas9 mutants were analyzed and show similar phenotypes seen in mutant mice recovered from the phenotype screen. A similar pattern of dysplasia and aplasia were seen in the cerebellum, olfactory bulb and hippocampus of mice recovered in the screen.

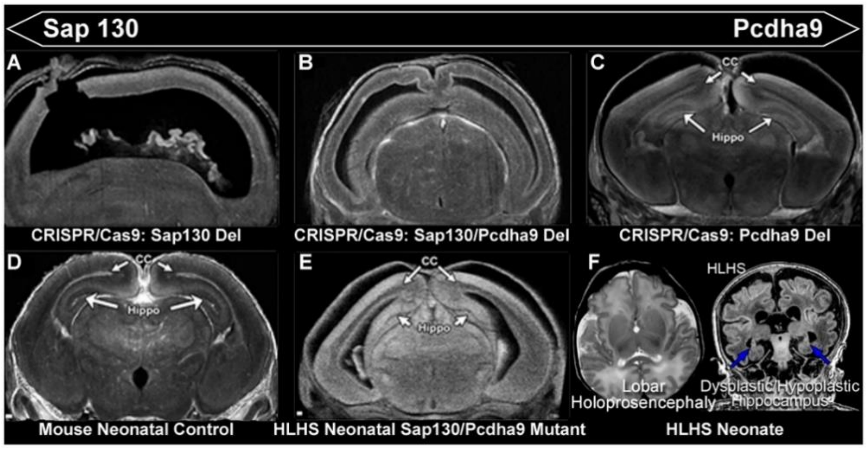

Supplemental Figure S9. Holoprosencephaly and Hippocampal Malformations Seen in Human and Mouse. A, Alobar holoprosencephaly seen in Sap130 CRISPR/Cas9 deletion mouse. B, Lobar holoprosencephaly seen in Sap130/Pcdha9 double CRISPR/Cas9 deletion mouse. C, Pcdha9 CRISPR/Cas9 deletion mouse displaying cerebral hypoplasia and dysmaturation as well as hippocampal abnormalities. D, Control mouse showing normal cortical and hippocampal anatomy. E, Sap130/Pcdha9 double mutant mouse exhibiting cortical, corpus collosum, and hippocampal abnormalities. F, Lobar holoprosencephaly and hippocampal malformations observed in human subjects with HLHS.
